# The polyamine transporter ATP13A3 mediates difluoromethylornithine‐induced polyamine uptake in neuroblastoma

**DOI:** 10.1002/1878-0261.13789

**Published:** 2025-02-21

**Authors:** Mujahid Azfar, Weiman Gao, Chris Van den Haute, Lin Xiao, Mawar Karsa, Ruby Pandher, Ayu Karsa, Dayna Spurling, Emma Ronca, Angelika Bongers, Xinyi Guo, Chelsea Mayoh, Youri Fayt, Arthur Schoofs, Mark R. Burns, Steven H. L. Verhelst, Murray D. Norris, Michelle Haber, Peter Vangheluwe, Klaartje Somers

**Affiliations:** ^1^ Laboratory of Cellular Transport Systems, Department of Cellular and Molecular Medicine KU Leuven Belgium; ^2^ Children's Cancer Institute, Lowy Cancer Research Centre UNSW Sydney Australia; ^3^ School of Clinical Medicine, UNSW Medicine & Health UNSW Sydney Australia; ^4^ Group for Neurobiology and Gene Therapy KU Leuven Belgium; ^5^ Leuven Viral Vector Core KU Leuven Belgium; ^6^ Aminex Therapeutics Aminex Therapeutics Inc. Kirkland WA USA; ^7^ Laboratory of Chemical Biology, Department of Cellular and Molecular Medicine KU Leuven Belgium; ^8^ UNSW Centre for Childhood Cancer Research UNSW Sydney Australia

**Keywords:** neuroblastoma, DFMO, ATP13A3, AMXT 1501, polyamine depletion, polyamine transport inhibitor

## Abstract

High‐risk neuroblastomas, often associated with *MYCN* protooncogene amplification, are addicted to polyamines, small polycations vital for cellular functioning. We have previously shown that neuroblastoma cells increase polyamine uptake when exposed to the polyamine biosynthesis inhibitor difluoromethylornithine (DFMO), and this mechanism is thought to limit the efficacy of the drug in clinical trials. This finding resulted in the clinical development of polyamine transport inhibitors, including AMXT 1501, which is presently under clinical investigation in combination with DFMO. However, the mechanisms and transporters involved in DFMO‐induced polyamine uptake are unknown. Here, we report that knockdown of ATPase 13A3 (*ATP13A3*), a member of the P5B‐ATPase polyamine transporter family, limited basal and DFMO‐induced polyamine uptake, attenuated *MYCN*‐amplified and non‐*MYCN*‐amplified neuroblastoma cell growth, and potentiated the inhibitory effects of DFMO. Conversely, overexpression of ATP13A3 in neuroblastoma cells increased polyamine uptake, which was inhibited by AMXT 1501, highlighting ATP13A3 as a key target of the drug. An association between high *ATP13A3* expression and poor survival in neuroblastoma further supports a role of this transporter in neuroblastoma progression. Thus, this study identified ATP13A3 as a critical regulator of basal and DFMO‐induced polyamine uptake and a novel therapeutic target for neuroblastoma.

## Graphical abstract

Polyamine homeostasis relies on a balance between extracellular uptake and intracellular biosynthesis. Cancer cells exhibit elevated polyamine levels, leading to strategies that inhibit biosynthesis using difluoromethylornithine. However, this inhibition often triggers increased polyamine uptake via unknown pathways. Our work on neuroblastoma cells show that targeting ATP13A3, a polyamine transporter, with the inhibitor AMXT 1501 or gene silencing, combined with difluoromethylornithine treatment, effectively inhibits neuroblastoma cell growth.

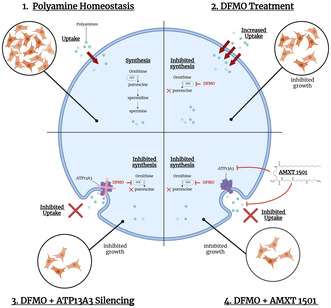

**Abbreviations**
AG: aminoguanidine
BDP: BODIPY
D498N: catalytically dead mutant
DFMO: difluoromethylornithine
FLUC: firefly luciferase
O.E.: overexpression
ORN: ornithine
PUT: putrescine
scr‐ctrl: scrambled control siRNA
SPD: spermidine
SPM: spermine
UQ: upper quartile
WT: wild‐type

## 1 Introduction

Neuroblastoma is the most common extracranial solid tumor in children that causes around 15% of childhood cancer‐related mortality [1, 2]. Despite intensified treatment, a substantial number of high‐risk patients do not respond well to conventional therapy, with more than half of children relapsing after therapy, the majority of whom ultimately die from the disease [3]. Moreover, survivors of high‐risk neuroblastoma often endure long‐term treatment‐related side effects, further demonstrating the pressing need for safer and more precise therapeutic strategies, which in turn requires more insight into underlying disease mechanisms to identify better therapeutic targets.

**Fig. 1:**
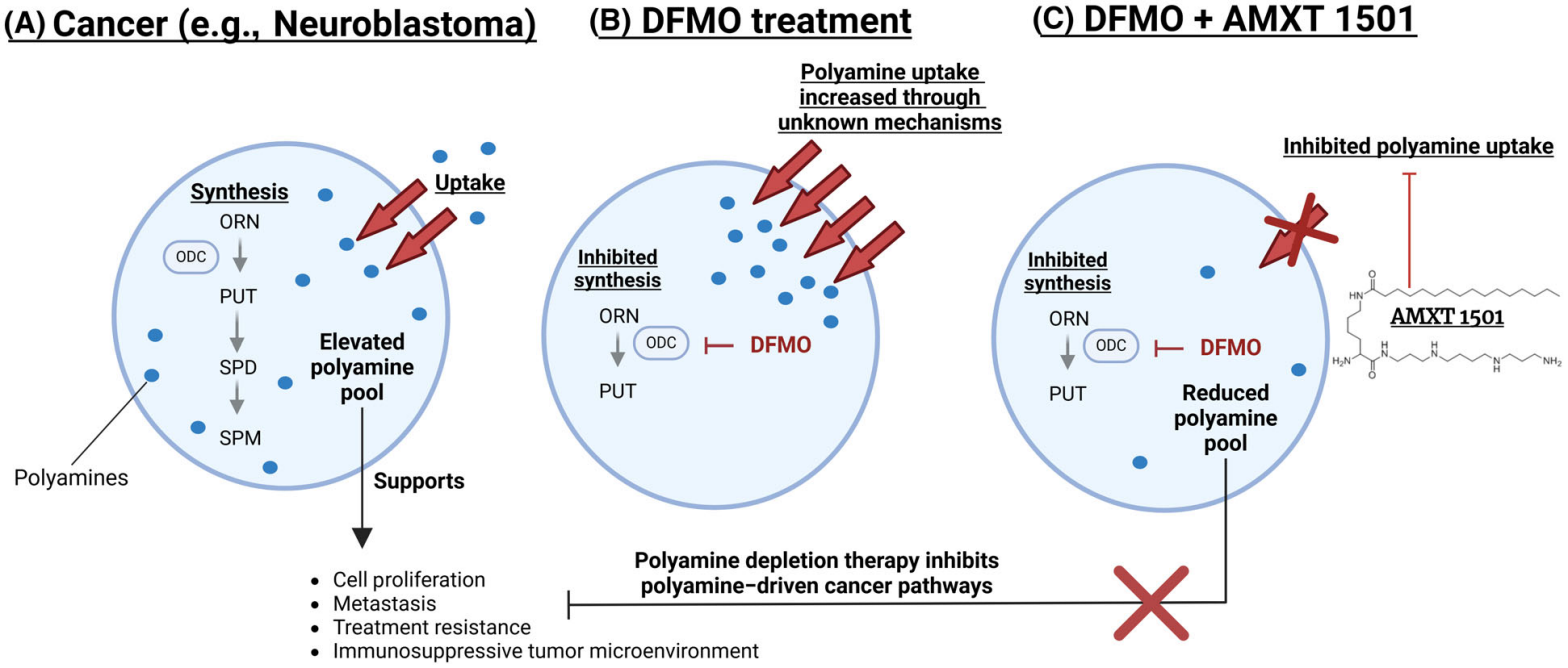
Polyamine homeostasis in cancer cells. (A) Cancer cells rely on an elevated polyamine pool to drive their hyperproliferative phenotypes. This resulted in efforts to reduce cancer cells' elevated polyamine pools by inhibiting synthesis using difluoromethylornithine (DFMO) to target the rate‐limiting biosynthetic enzyme ODC1. (B) Cancer cells rescue their polyamine levels by a compensatory increase in polyamine uptake, the mechanism of which is poorly understood. (C) Combined inhibition of both polyamine biosynthesis using DFMO and polyamine uptake using a polyamine transport inhibitor like AMXT 1501 reduces the intracellular polyamine pool in cancer cells, thereby inhibiting polyamine‐driven cancer pathways. Figure made using BioRender. DFMO, difluoromethylornithine; ODC1, ornithine decarboxylase; ORN, ornithine; PUT, putrescine; SPD, spermidine; SPM, spermine.

One therapeutic strategy currently under investigation for cancer is targeting its increased dependency on polyamines. As the three main polyamines in mammalian cells, putrescine, spermidine, and spermine are regulators of cell growth, proliferation, and survival, cancer cells are often characterized by elevated polyamine levels to sustain their hyperproliferative state [4, 5]. Cellular polyamine homeostasis is tightly regulated by fine‐tuning *de novo* synthesis, uptake from the extracellular environment, intracellular trafficking, and catabolism (Fig. 1). Neuroblastoma is characterized by increased levels of intracellular polyamines, as well as elevated expression of ornithine decarboxylase 1 (ODC1), the rate limiting enzyme in the *de novo* polyamine synthesis pathway, which is predictive of poor outcome [6]. However, while the polyamine synthesis inhibitor DFMO, which targets ODC1, appeared promising in reducing relapse and increasing overall survival time as maintenance therapy for high‐risk neuroblastoma in remission, it was not very effective in the relapsed setting [7, 8, 9]. In exploring the disappointing results of DFMO, we previously reported that neuroblastoma cells under a DFMO treatment regime compensate for reduced intracellular polyamine content by upregulating uptake of extracellular polyamines (Fig. 1) [10]. We and others demonstrated that combination therapy that uses polyamine transport inhibitors such as AMXT 1501 in conjunction with DFMO increases DFMO efficacy [10, 11, 12]. These results in preclinical models culminated in phase 1B/2A clinical trials for the combination of AMXT 1501 and DFMO in adult cancer patients (NCT03536728; NCT05500508). However, the molecular identity of the polyamine transporter(s) that is (are) targeted by AMXT 1501 and is (are) responsible for the DFMO‐induced polyamine uptake remains unknown.

We previously uncovered a putative role for SLC3A2 in polyamine transport in neuroblastoma [10]. We also established that members of the P5B‐ATPase family (ATP13A2‐4) are major polyamine transporters in the polyamine transport system [13, 14] that may be implicated in cancer [15, 16, 17], but their relative role in neuroblastoma remains unknown. In this study, we discovered that high ATP13A3 expression is associated with worse overall and event‐free survival in neuroblastoma patients and that ATP13A3, but not SLC3A2, is a primary mediator of DFMO‐induced polyamine uptake in this disease. Targeting ATP13A3, either by AMXT 1501 treatment or by silencing its expression, not only inhibits basal and DFMO‐induced compensatory polyamine uptake and reduces the intracellular polyamine pool, but also abrogates colony formation capacity and growth of neuroblastoma cells while increasing the cells' sensitivity to DFMO. Collectively our findings underpin ATP13A3 as not only a primary driver of DFMO‐induced compensatory polyamine uptake in neuroblastoma but also a candidate target of AMXT 1501.

## 2 Materials and methods

### 2.1 Reagents

Polyamines putrescine dihydrochloride (P7505), spermidine (S2626), and spermine (3256) were purchased from SIGMA, Darmstadt, Germany. All polyamines were dissolved to a final stock concentration of 500 mm (200 mm in the case of spermine) in 0.1 m MOPS‐KOH (pH 7.0). AMXT 1501 was kindly provided by Aminex Therapeutics. Antibiotics (puromycin and blasticidin, INVIVOGEN, San Diego, California, USA) were used as selection agents after SH‐SY5Y cells were transduced. Penicillin–streptomycin (P4458, Merck, Darmstadt, Germany) was added to culture medium for cell culturing. Antibiotics were removed from cells used in experiments.

### 2.2 Constructs and cell lines

SH‐SY5Y cells (CRL‐2266, RRID:CVCL_0019) were purchased from ATCC, KELLY cells (ACC354, RRID:CVCL_2092) cells from DSMZ, BE(2)‐C cells (CRL‐2268, RRID:CVCL_0529) from ATCC, Tet‐21/N cells (RRID:CVCL_9812) were a gift from Manfred Schwab, German Cancer Research Centre, Heidelberg, Germany. All utilized cell lines were mycoplasma‐free and have been authenticated in the past 3 years by STR profiling. To produce stable cell lines either overexpressing wild‐type (WT) human ATP13A3, a catalytically dead D498N ATP13A3 mutant, human FLAG‐tagged SLC3A2 isoform c or Firefly Luciferase (FLUC) cells were subjected to lentiviral transduction as previously described [18]. Generation of stable ATP13A3 knockdown cell lines was achieved using validated microRNA (miR) based short‐hairpin lentiviral vectors targeting three different regions: mirKD1: AATCACAACAGATTCGTTATTT; mirKD‐2: TCAATCGTAAGCTCACTATATT; mirKD‐3: AGACCACCTTCGGGTCTTATAT. miRNA targeting Firefly Luciferase (mirFLUC: ACGCTGAGTACTTCGAAATGTC) was used as a negative control. Following transduction, the cells were kept under selection (2 μg·mL^−1^ puromycin for overexpression models or 5 μg·mL^−1^ blasticidin for the KD models). For siRNA knockdown of SLC3A2 and ATP13A3, multiple independent siRNAs were purchased from GE Dharmacon™, and following optimization, the siRNAS that gave the greatest knockdown were used for further experiments. The sequences of the siRNAs are the following: ATP13A3 siKD‐1: GGUCAUAAUUAUCGAGUCU; ATP13A3 siKD‐2: AGUCUUCUCUCGUAGGUUA; ATP13A3 siKD‐3: AGAAACACAUAAACGACAU; ATP13A3 siKD‐4: CAAUUGACCCAGAGGCUAU; SLC3A2 siKD‐1: GGACCUUACUCCCAACUAC; SLC3A2 siKD‐2: GUUCCAGGUUCGGGACAUA. siRNAs were transfected into cells using RNAiMAX according to manufacturer's instructions. The parental SH‐SY5Y and BE(2)‐C cells were grown in 75 cm^2^ cell culture flasks in Dulbecco's Modified Eagle's Medium (DMEM) + 10% fetal bovine serum (FBS) (Life Technologies, Carlsbad, California, USA), and stable ATP13A3 knockdown and overexpression SH‐SY5Y cells were cultured in DMEM with high glucose supplemented with sodium bicarbonate, sodium pyruvate (SIGMA), Penicillin/streptomycin, L‐Glutamine and 10–15% FBS. KELLY and Tet‐21/N cells were cultured in RPMI medium with 10–15% FBS. Cells were maintained at 5% CO_2_ and 37 °C and were split at full confluency using trypsin ethylenediaminetetraacetic acid solution (SIGMA) and phosphate‐buffered saline (PBS) solution (SIGMA) without magnesium and calcium. The number of passages did not exceed 25.

### 2.3 Western blotting

Total cell lysates were made in RIPA buffer (Thermo Fisher, Waltham, Massachusetts, USA, 89900) containing protease inhibitors (Sigma‐Aldrich, Darmstadt, Germany, Cat# S8830). The protein concentration was determined using the Pierce BCA Protein Assay Kit (Thermo Fisher, Cat# 23227). Primary antibodies for ATP13A3 (Sigma‐Aldrich, Cat# HPA029471, RRID: AB_10600784), SLC3A2 (Bio‐Rad, Hercules, California, USA, Cat# VPA00372, RRID:AB_3662945), actin (Sigma‐Aldrich, Cat# A2066, RRID:AB_476693) and GAPDH (Sigma‐Aldrich, Cat# G8795, RRID:AB_1078991) were diluted 1 : 1000 or 1 : 5000 respectively, in 1% BSA/TBS‐tween and incubated overnight at 4 °C. Secondary antibodies, IgG HRP‐linked Anti‐rabbit (Cell Signaling, Danvers, Massachusetts, USA, Cat# 7074S, RRID:AB_2099233) and IgG HRP‐linked Anti‐mouse (Cell Signaling, Cat# 7076S, RRID:AB_330924), were diluted in 5% milk/TBS‐tween solution (1 : 5000) and added for 1 h. SuperSignal™West Pico PLUS chemiluminescent Substrate (Thermo Fisher, Cat# 34580) was used to detect bands, utilizing the Bio‐Rad Chemidoc™ MP imaging system. Densitometry measurements were performed with imagej, LOCI, University of Wisconsin. Densitometry data were first normalized to the signal obtained for Actin or GAPDH to control for total protein loading, followed by the calculation of changes in expression relative to control conditions.

### 2.4 PCR

RNA was isolated with RNeasy Micro kit (Qiagen, VIC, Australia). Quantitation of RNA was performed using NanoDrop™ 2000c Spectrophotometers (Thermo Scientific). Reverse transcription was performed with iScript™ Advanced cDNA Synthesis Kit (BioRad) and the PCR was excuted with PrimePCR SYBR^®^ Green Assays (BioRad) on a QuantStudio™ 3 Real‐Time PCR System (Thermo Fisher Scientific, Waltham, Massachusetts, USA). Forward primer sequence for ATP13A3: TACTGTGGAGCACTGATG; Reverse primer sequence for ATP13A3: GAGTTGCCACCATGTCATGC. Forward primer sequence for ATP13A2: ACCGGTTATGGGACCCTGAC; Reverse primer sequence for ATP13A2: GTGATAGCCGATGACCCTCC. Forward primer sequence for SLC3A2: GGACCTTACTCCCAACTACCG; Reverse primer sequence for SLC3A2: TCCAGAGCATCCTTCACCTTG.

### 2.5 Cytotoxicity (viability) assays

The 4‐methylumbelliferyl heptanoate (MUH) assay was performed as previously described [13]. Assays were in parallel executed in the presence of aminoguanidine (1 mm) to eliminate potential cytotoxic effects of by‐products generated by serum amine oxidases present in culture medium supplemented with FBS on extracellular polyamines [19, 20]. For MUH assays involving AMXT 1501, the cells were pretreated overnight. Subsequent treatment with polyamines was performed in the presence of AMXT 1501.

### 2.6 Metabolomics

Intracellular polyamine levels were measured by the KU Leuven metabolomics core using LCMS. The samples were prepared using previously described protocols [13].

### 2.7 BODIPY‐polyamine uptake assays

Polyamine‐BODIPY (polyamine‐BDP) uptake was measured by flow cytometry as previously described [21]. For uptake assays involving AMXT 1501, the cells were pretreated overnight. Subsequently, polyamine‐BDPs were added in the presence of AMXT 1501. 1 mm aminoguanidine was supplied in the medium to block serum amine oxidases in culture medium supplemented with FBS [19, 20].

### 2.8 Radio‐labeled polyamine uptake assays

^3^H‐Spermidine Trihydrochloride (NET522001MC, 16.6 Ci·mmol^−1^; Perkin Elmer, Waltham, Massachusetts, USA) or ^3^H‐Putrescine Trihydrochloride (ART‐0279‐1mCi, 60 Ci·mmol^−1^; American Radiolabeled Chemicals) were added to cells at 0.1–0.2 μCi (1.67–3.33 μm), concentrations that obey the Michaelis Menten kinetics, and incubated at 37 °C for 60–90 min prior to washing and lysis. Radiolabeled polyamine uptake incorporation was determined by scintillation counting and normalized to protein concentration as previously described [10].

### 2.9 Cell growth assays

Cells were seeded in a 12‐well plate at 15 000 cells per well and transfected with siRNA at 24 h postseeding, followed by media replacement with or without drug treatment. Phase contrast confluence cell images were obtained using a 10× objective lens every 6 h for 168 h and the average confluence of each well was calculated with the IncuCyte S3 Live Cell Analysis System (Sartorius, Göttingen, Germany).

### 2.10 Colony formation assays

Cells were plated in six‐well plates at a density of 500–750 cells per well, and 24 h after seeding were treated with a serial dilution of DFMO and/or AMXT1501 for 72 h. Assays were in parallel executed in the presence of 0.5 mm aminoguanidine. After 10–14 days, cells were fixed and stained, and colonies counted using the imagej software. Each experiment was performed in duplicate or triplicate.

### 2.11 Synergy assays

For synergy viability assays, cells were seeded at 5000–10 000 cells per well for 24 h before being treated with increasing doses of drugs in a 6 × 6 combination matrix format. Cell viability was measured by resazurin reduction‐based assays after a 3‐day treatment. Synergy was scored according to the Bliss Independence model [22] and visualized by Combenefit [23].

### 2.12 ChIP‐seq data processing and analysis

ChIP‐seq fastq file data sets of MYCN in KELLY, BE(2)‐C and NGP (RRID: RRID:CVCL_2141) cell lines (GSE80151) were obtained, as well as for c‐MYC in NB69 cell line (GSE138295, RRID:CVCL_1448). Quality control analysis of sequencing reads was performed using fastqc (v0.11.9, https://www.bioinformatics.babraham.ac.uk/projects/fastqc/) and cutadapt (v4.2, https://journal.embnet.org/index.php/embnetjournal/article/view/200) was applied for adapter trimming. Reads were aligned against the GRCh38 human reference genome using bowtie2 (v2.5.0, https://pubmed.ncbi.nlm.nih.gov/22388286/). BAM files were sorted and duplicates marked using picard (v2.27.5, http://broadinstitute.github.io/picard/) and samtools (v1.12, https://academic.oup.com/gigascience/article/10/2/giab008/6137722?login=false). Peak calling was performed using macs2 (v 2.2.7.1, https://genomebiology.biomedcentral.com/articles/10.1186/gb‐2008‐9‐9‐r137), with narrow peak calling run with ‐‐keep‐dup auto and ‐p 1e‐9 for MYCN and ‐q 0.05 for c‐MYC. chipseeker (v1.36.0) was employed for peak annotation with default parameters and the TxDb.Hsapiens.UCSC.hg38.knownGene transcript database. BigWig coverage files were generated using deeptools bamcoverage (v3.5.1, https://academic.oup.com/nar/article/44/W1/W160/2499308). Phred quality scores with a threshold of q30 were used to eliminate false positives.

### 2.13 Multivariate cox regression analysis

The Kocak microarray dataset (*N* = 649) and SEQC RNA‐seq dataset (*N* = 498) were accessed through the R2: Genomics Analysis and Visualization Platform (R2: Genomics Analysis and Visualization Platform (amc.nl)). Event‐free and overall survival data for the KOCAK dataset were supplied by the University of Cologne, Department of Pediatric Oncology and Hematology with clinical data such as patient age, tumor stage and *MYCN* amplification status retrieved from the Gene Expression Omnibus database (GSE45480). Kaplan–Meier survival curves were generated in r, and multi‐variate analysis was performed with a cox proportional hazard model (using coxph function in r). *ATP13A3* expression was separated into either high (top 25th percentile) or low (bottom 75th percentile) and patient's age into either ≥ 18 years or < 18 years. The SEQC dataset covariates were *ATP13A3* expression (high *versus* low), age (≥ 18 *versus* < 18 months), and *MYCN* status (amplified *versus* nonamplified). Kocak covariates also included tumor stage (stage 3/4 versus stage 1/2/4S).

### 2.14 Statistics

Data are presented as mean ± SEM of three independent repeats (unless otherwise stated). All statistical analyses were performed with graphpad prism 9.5.1(733, San Diego, California, USA). Statistical significance was determined by one‐ or two‐way ANOVA followed by multiple comparisons using Dunnett's/Tukey's *post hoc* tests. For datasets comparing two groups, unpaired *t*‐tests or one sample *t*‐tests were performed. Statistical significance was defined as *P < 0.05, **P < 0.01, ***P < 0.001, ****P < 0.0001 or ns (nonsignificant).

## 3 Results

### 3.1 High *ATP13A3* and *SLC3A2* expression are prognostic of inferior outcome in neuroblastoma

**Fig. 2:**
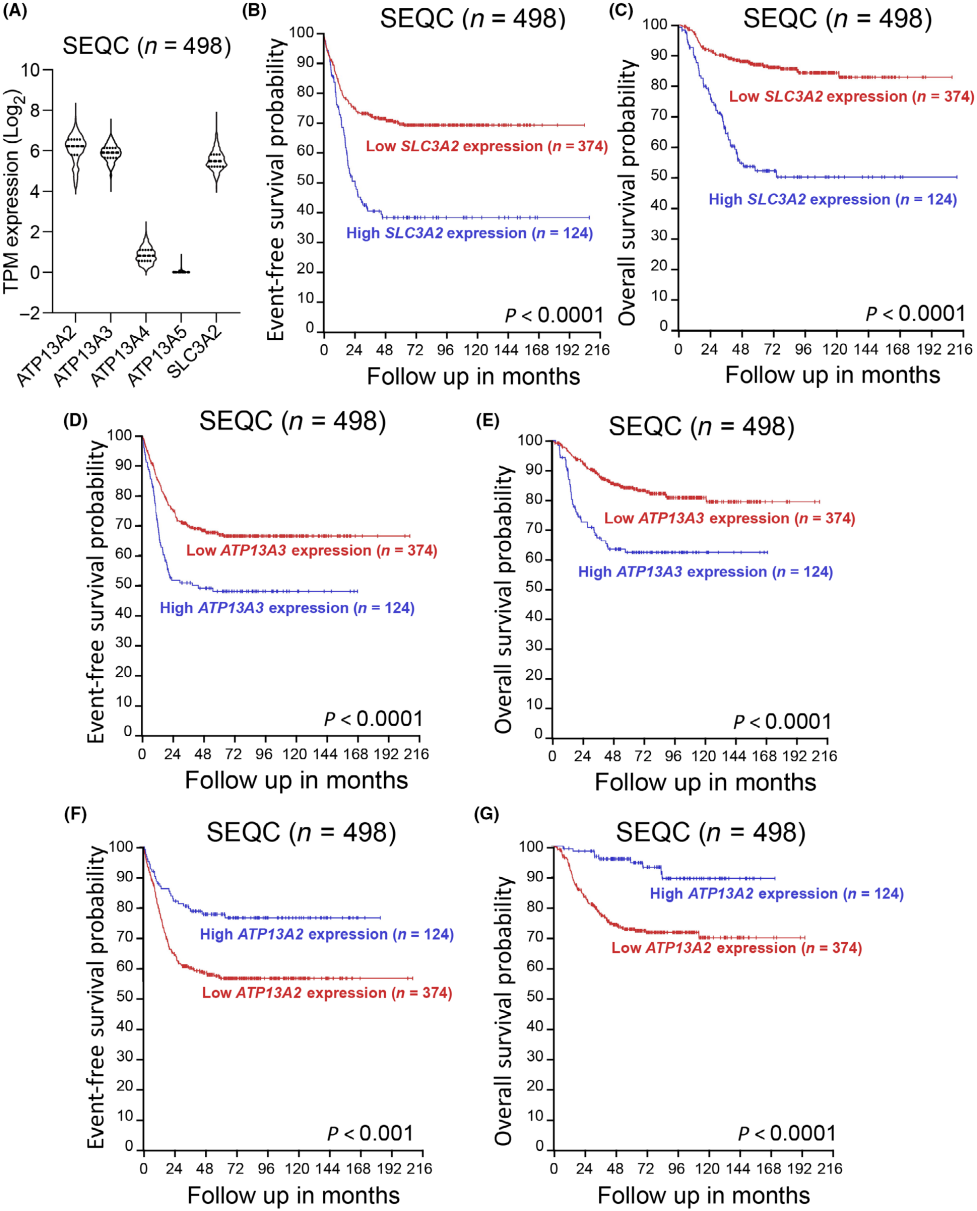
High expression of *SLC3A2* or *ATP13A3* is a prognostic predictor for inferior outcome in neuroblastoma. (A) mRNA expression (Transcripts Per Million, TPM) of polyamine transporters in the SEQC neuroblastoma database (*n* = 498). Graph represents the distribution of data density of gene expression in neuroblastoma patients, with the central line representing the median, and the upper and lower lines indicating the third (Q3) and first (Q1) quartiles. (B–G) Kaplan–Meier survival curves for *SLC3A2* (B, C), *ATP13A3* (D, E) and *ATP13A2* (F, G) in the neuroblastoma SEQC cohort. Patients were dichotomized around the upper quartile (UQ) of gene expression. Log‐rank test was used to compare the survival curves of the high and low expression groups.

We previously identified SLC3A2 as a candidate polyamine transporter in neuroblastoma cells [10] and established members of the P5B‐ATPase family, as key components of the mammalian polyamine transport system [13, 14, 17]. To investigate the biological significance of these transporters in neuroblastoma, we first assessed and compared their expression in a publicly available neuroblastoma patient cohort (SEQC, *n* = 498) using the R2: Genomics Analysis and Visualization Platform (https://r2.amc.nl). *SLC3A2*, *ATP13A2*, *ATP13A3* are abundantly expressed in neuroblastoma tumors, while *ATP13A4* and *ATP13A5* expression is low (Fig. 2A). Survival analyses in the SEQC cohort confirmed our prior findings that elevated expression of *SLC3A2* is associated with worse event‐free and overall survival in neuroblastoma (Fig. 2B,C). Similarly, high *ATP13A3* expression was correlated with poorer outcome in two neuroblastoma cohorts (Fig. 2D,E, Fig. S1), while for *ATP13A2* the opposite was observed (Fig. 2F,G, Fig. S2). No consistent correlations were found between *SLC3A2*, *ATP13A3* or *ATP13A2* expression levels (data not shown).

### 3.2 SLC3A2‐mediated polyamine transport does not contribute to DFMO‐induced compensatory polyamine uptake

**Fig. 3:**
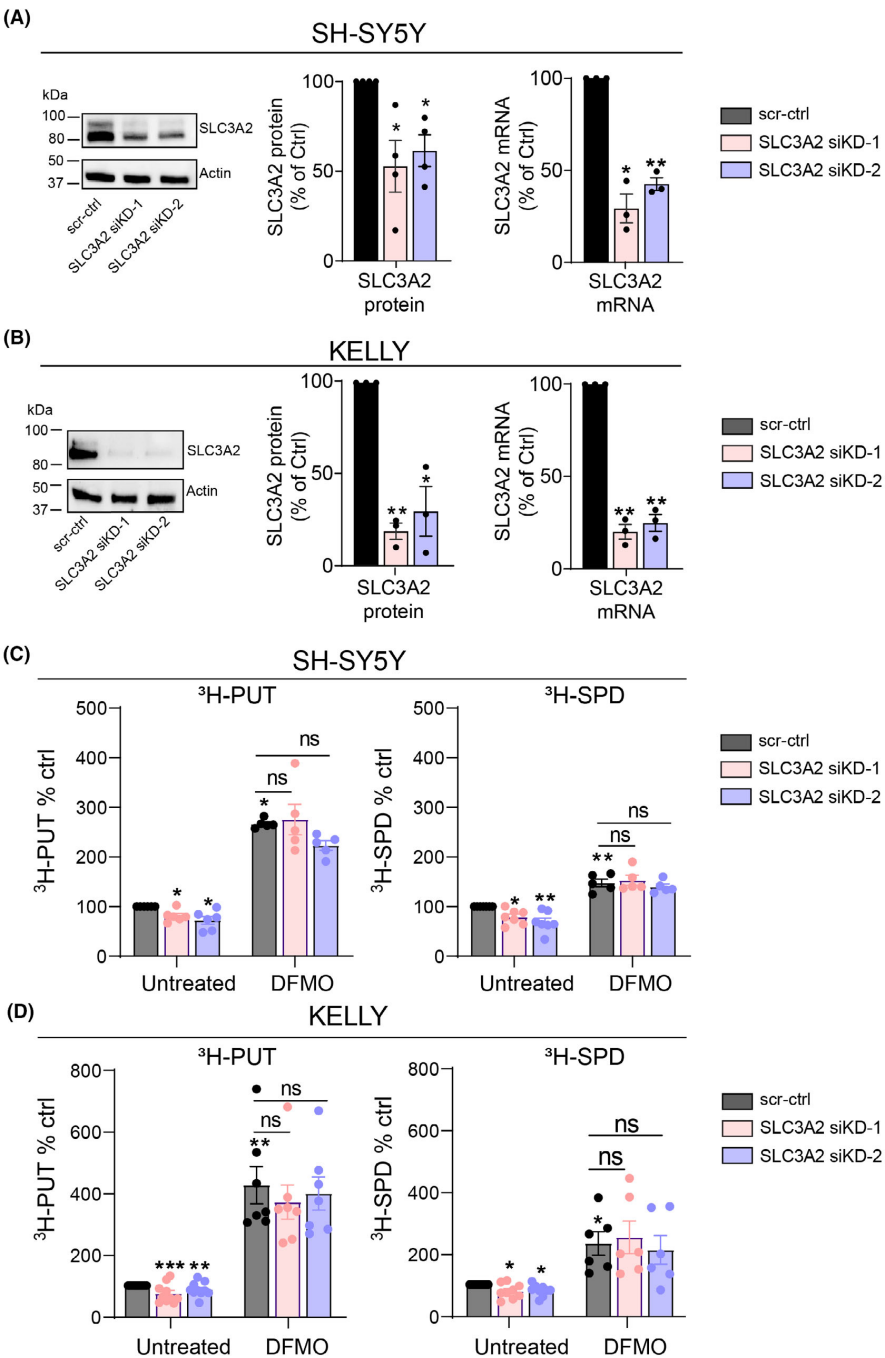
SLC3A2 silencing does not abrogate DFMO‐induced polyamine uptake in neuroblastoma cells. (A, B) Reduced SLC3A2 protein (left) and mRNA (right) levels after siRNA‐mediated silencing (for 48 h) in SH‐SY5Y (A) and KELLY (B) cells. One sample *t*‐test was used to assess the significance of silencing relative to scr‐ctrl transduced cells. Presented immunoblots are representative of results obtained in at least three independently performed experiments. Graphs depict mean ± SEM of at least three independent biological repeats (*n* = 4 for SH‐SY5Y ATP13A3 protein; *n* = 3 for SH‐SY5Y ATP13A3 mRNA and KELLY ATP13A3 protein and mRNA). (C, D) Measurement of radiolabeled putrescine (PUT) or spermidine (SPD) uptake after SLC3A2 silencing with two different siRNAs (siKD‐1, siKD‐2) *versus* a scrambled control siRNA (scr‐ctrl). One sample *t*‐test was used to assess the significance of the changes in polyamine uptake *versus* untreated cells transfected with scr‐ctrl (100%) which is indicated by the asterisks on top of the bars. Comparisons between the uptake % in all difluoromethylornithine (DFMO)‐treated groups were made by one‐way ANOVA, and represented by asterisks above connecting lines between bars. All comparisons between DFMO‐treated groups were found to be non‐significant (ns). Graphs depict mean ± SEM of at least five independent biological replicates (*n* = 6 for SH‐SY5Y PUT untreated; *n* = 5 for SH‐SY5Y PUT DFMO; *n* = 7 for SH‐SY5Y SPD untreated; *n* = 5 for SH‐SY5Y SPD untreated; *n* = 10 for KELLY PUT untreated; *n* = 7 for KELLY PUT DFMO; *n* = 9 for KELLY SPD untreated; *n* = 6 for KELLY SPD DFMO). Statistical significance was defined as *P < 0.05, **P < 0.01, ***P < 0.001 or ns (non‐significant). DFMO, difluoromethylornithine.

To examine which polyamine transporters play a role in basal and DFMO‐induced polyamine uptake, we started by confirming our earlier discovery that SLC3A2 contributes to polyamine uptake in *MYCN*‐amplified neuroblastoma cells [10]. We silenced *SLC3A2* expression in non‐*MYCN*‐amplified (SH‐SY5Y) as well as *MYCN*‐amplified (KELLY) neuroblastoma cells and subsequently measured putrescine and spermidine uptake. siRNA‐mediated silencing significantly decreased *SLC3A2* mRNA expression in both neuroblastoma cell lines 48–72 h after transfection (Fig. 3A,B, Fig. S3), which attenuated putrescine and spermidine uptake levels (Fig. 3C,D).

In light of the finding that neuroblastoma cells treated with DFMO compensate for a decrease in intracellular polyamines by augmenting polyamine uptake, we next assessed whether SLC3A2‐mediated polyamine uptake contributes to the DFMO‐induced increase in polyamine uptake [10, 24, 25]. We first confirmed that 1 mm of DFMO treatment significantly increased the uptake of radiolabeled putrescine and spermidine in both non‐*MYCN* amplified and *MYCN*‐amplified neuroblastoma cells (Fig. 3C,D). However, knockdown of SLC3A2 did not significantly abrogate the DFMO‐induced increase in polyamine uptake in *MYCN*‐amplified (KELLY) or non‐*MYCN*‐amplified (SH‐SY5Y) cells (Fig. 3C,D), indicating that SLC3A2 is not the main driver for this phenomenon. Together with the observation that high ATP13A3 expression is predictive of poor outcome in neuroblastoma (Fig. 2D,E), this finding provided impetus to subsequently investigate the potential of ATP13A3 as a contributor to polyamine transport in neuroblastoma and to polyamine uptake mechanisms that limit responsiveness to DFMO.

### 3.3 ATP13A3 mediates polyamine transport in neuroblastoma cells

**Fig. 4:**
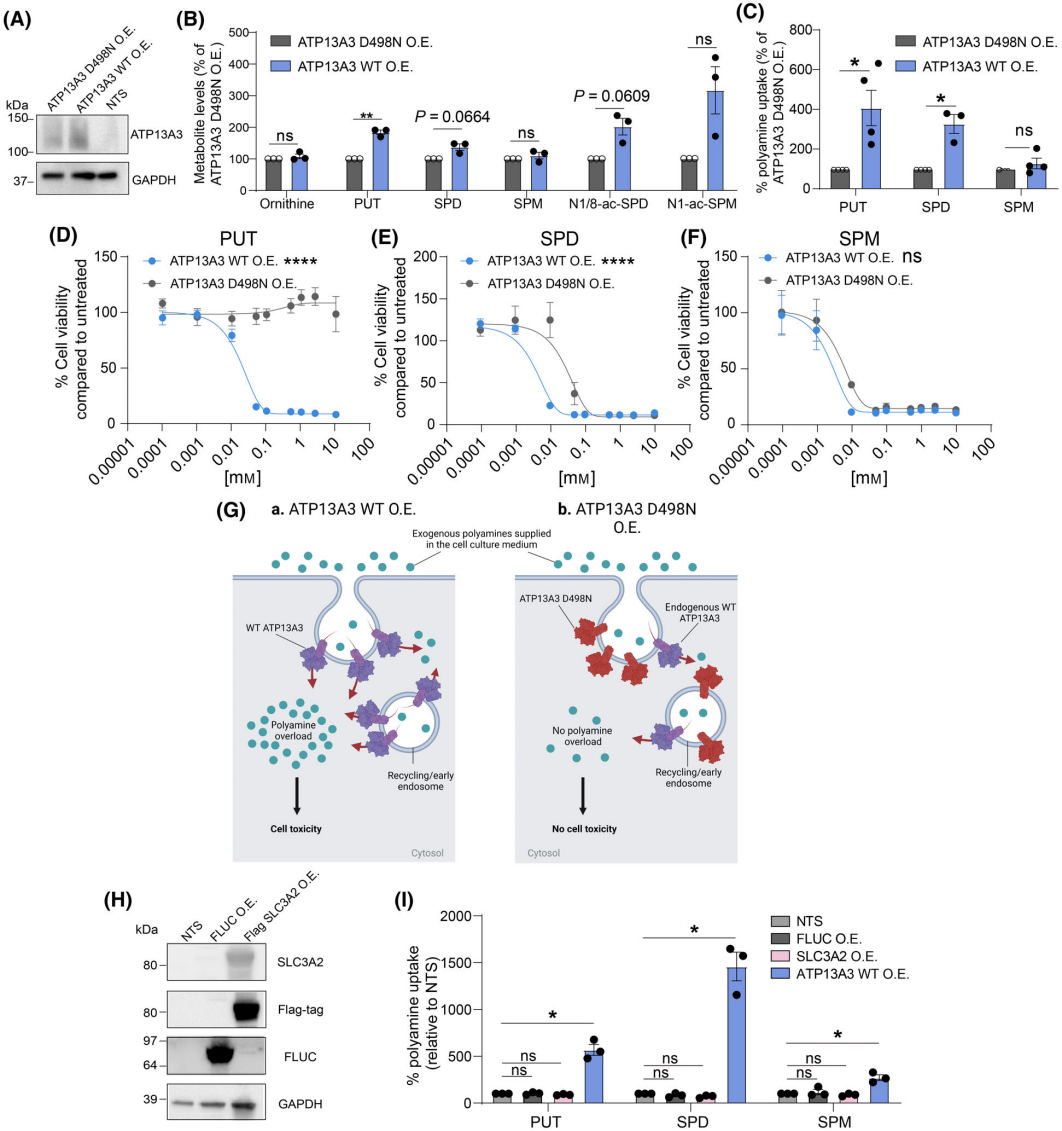
ATP13A3 mediates polyamine uptake in neuroblastoma cells. (A) Western blot showing ATP13A3 protein levels in the SH‐SY5Y ATP13A3 wild type overexpression (ATP13A3 WT O.E.) or ATP13A3 D498N overexpression (ATP13A3 D498N O.E.) models *versus* the nontransduced (NTS) control. Representative blots from three independent biological replicates are shown. (B) Mass spectrometry‐based measurement of metabolites in the polyamine pathway of the SH‐SY5Y ATP13A3 cell models (putrescine, PUT; spermidine, SPD; spermine, SPM; acetylated spermidine, N1/8‐ac‐SPD; and acetylated spermine, N1‐ac‐SPM). One sample *t*‐test was used for statistical analysis. Graphs depict mean ± SEM of three independent biological replicates. (C) Cellular uptake of fluorescent BODIPY (BDP)‐conjugated PUT, SPD and SPM in SH‐SY5Y ATP13A3 cell models. One sample *t*‐test was used to determine the significance of the difference in uptake. Graphs depict mean ± SEM of at least three independent biological replicates (*n* = 4 for PUT and SPM, *n* = 3 for SPD). (D–F) Cell viability assay (MUH reagent) in SH‐SY5Y ATP13A3 overexpression cells exposed to toxic effects of exogenously added PUT, SPD and SPM. Two‐way ANOVA was used for statistical analysis. Graphs depict mean ± SEM of at least two independent biological replicates (PUT, *n* = 3; SPD, *n* = 3; SPM, *n* = 2). (G) Overexpression of (a) ATP13A3 WT in SH‐SY5Y cells leads to increased uptake of exogenous polyamines inducing cell toxicity. No toxicity is observed by overexpressing (b) ATP13A3 D498N, where the uptake of extracellular polyamines is driven solely by endogenously present polyamine transporters. Figure made using BioRender. (H) Western blot showing the successful overexpression of Flag‐tagged SLC3A2 and Firefly Luciferase (FLUC) O.E. in SH‐SY5Y cells. Presented immunoblots are representative of results obtained in three independently performed experiments. (I) Uptake of BDP‐conjugated polyamines in SH‐SY5Y cells overexpressing SLC3A2 *versus* ATP13A3 overexpressing cells. 1 mm of aminoguanidine was added to cell culture medium. Graphs depict mean ± SEM of at three independent biological replicates. One sample *t*‐test was used to assess the significance of the changes in polyamine uptake *versus* NTS cells (100%) which is indicated by the asterisks on top of the bars. Comparisons between the uptake % in all overexpression groups were made by one‐way ANOVA, and represented by asterisks above connecting lines between bars. Statistical significance was defined as *P < 0.05, **P < 0.01, ****P < 0.0001 or ns (non‐significant).

To study the role of ATP13A3 in polyamine uptake and homeostasis, we first generated SH‐SY5Y cells that overexpress wild‐type (WT) ATP13A3 and ATP13A3 D498N—a catalytically dead/transport deficient mutant in which the catalytic site for reversible auto‐phosphorylation (Asp498) is replaced by asparagine (Fig. 4A). ATP13A3 overexpression did not markedly change the expression of *SLC3A2* or *ATP13A2* (Fig. S4). Interestingly, we observed that cells overexpressing ATP13A3 WT exhibited significantly higher basal intracellular levels of putrescine, and a trend toward higher spermidine content compared to cells overexpressing ATP13A3 D498N (Fig. 4B, Table S1). The levels of acetylated spermidine and spermine, by‐products of polyamine degradation, were also increased in ATP13A3 WT overexpressing cells as compared to cells with ATP13A3 D498N overexpression. To demonstrate that the observed higher polyamine levels were due to increased ATP13A3‐mediated polyamine uptake [14, 16], we assessed the uptake of fluorescent BODIPY (BDP)‐conjugated polyamines, that behave similarly to radiolabeled polyamines [21]. We noted a significant increase in putrescine‐BDP and spermidine‐BDP, but not in spermine‐BDP uptake upon ATP13A3 WT overexpression compared to ATP13A3 D498N overexpression (Fig. 4C).

To cross‐validate the polyamine transport function of ATP13A3 in neuroblastoma cells, we assessed the effect of ATP13A3 overexpression on mediating the cytotoxic effects of high intracellular polyamine levels. Despite being essential for cell physiology and survival, at high intracellular concentrations, polyamines are cytotoxic, and we utilized this phenomenon to provide further evidence for ATP13A3 functioning as a transporter mediating polyamine uptake into neuroblastoma cells [13]. As an independent, but indirect measurement of polyamine uptake activity, we observed that upon extracellular polyamine supplementation to the culture medium, ATP13A3 WT overexpression, but not ATP13A3 D498N overexpression, increased the sensitivity of SH‐SY5Y cells to increasing doses of exogenous putrescine and spermidine, while a similar trend was observed for spermine albeit not statistically significant (Fig. 4D–F). As serum amine oxidases present in serum added to culture medium can result in the formation of toxic by‐products of extracellular polyamines, we performed these experiments also in the presence of 1 mm aminoguanidine, which inhibits amine oxidases, confirming that the relative polyamine uptake window between ATP13A3 WT and ATP13A3 D498N SH‐SY5Y cells (Fig. 4C) is not impacted by extracellular serum amine oxidase activity (Fig. S5A–C), and demonstrating that the observed toxicity of exogenously added polyamines (Fig. 4D–F) in SH‐SY5Y cells overexpressing ATP13A3 WT in these supplementation assays does not stem from extracellular polyamine degradation by serum amine oxidase (Fig. S5D–F). Thus, ATP13A3 WT overexpression confers higher polyamine‐induced toxicity to neuroblastoma cells as a consequence of excessive polyamine uptake (Fig. 4Ga–b).

To directly compare the relative impact of SLC3A2 and ATP13A3 on cellular polyamine uptake, we also generated stable SH‐SY5Y cell lines overexpressing SLC3A2 or Firefly Luciferase (FLUC, a negative control for transduction) (Fig. 4H). Unlike ATP13A3, SLC3A2 overexpression is not sufficient to induce increased uptake of any of the BDP‐labeled polyamines (Fig. 4I).

**Fig. 5:**
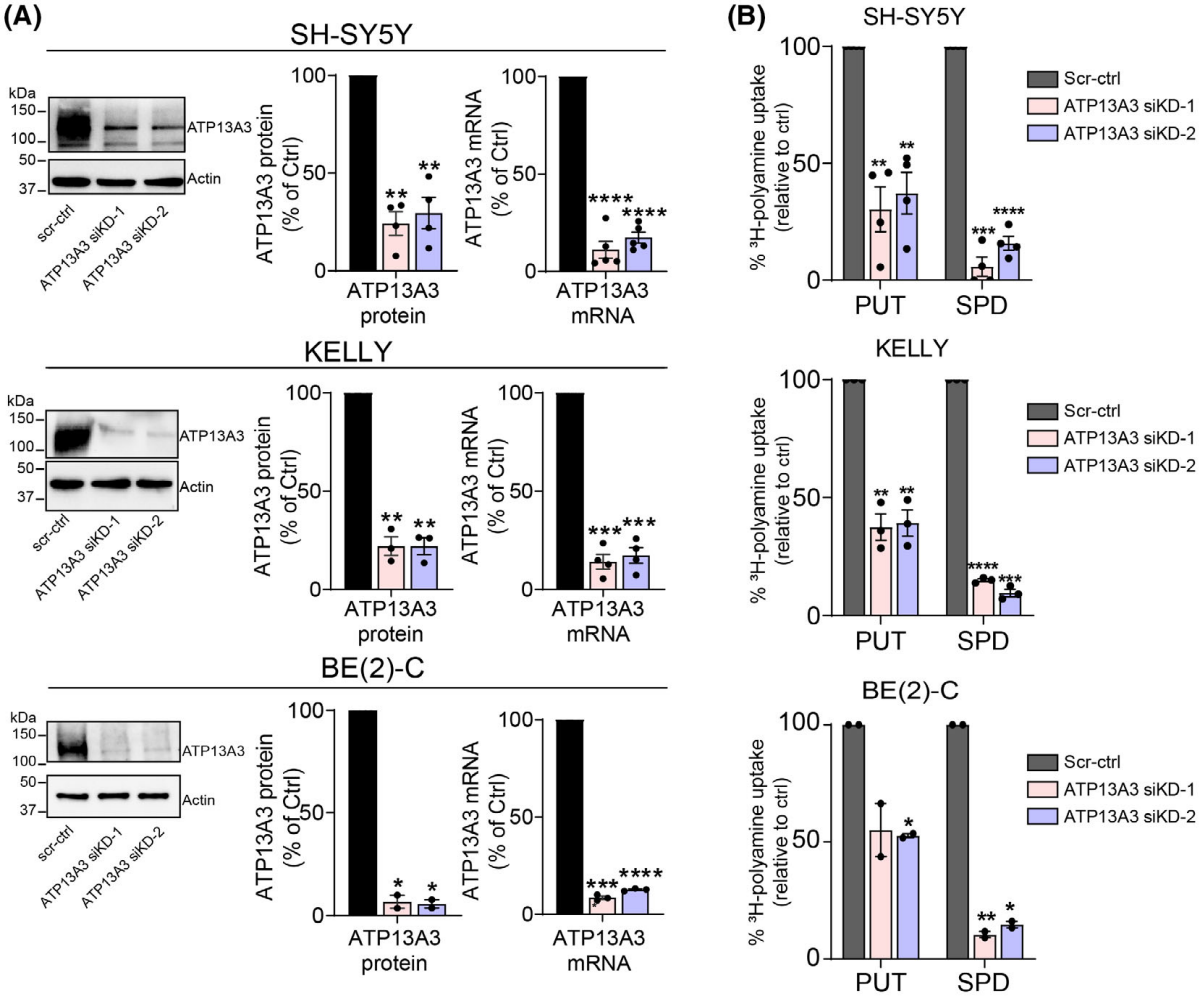
ATP13A3 knockdown reduces polyamine uptake in multiple neuroblastoma cell lines. (A) Reduced ATP13A3 protein (left) and mRNA (right) levels after siRNA‐mediated silencing in various neuroblastoma cell lines (48 h). One sample *t*‐test was used to assess the significance of silencing relative to scrambled control (scr‐ctrl) transduced cells. The depicted blot is representative for data obtained in at least two independently performed experiments (SH‐SY5Y, *n* = 4; KELLY, *n* = 3; BE(2)‐C, *n* = 2). mRNA levels were quantified and represented as mean ± SEM of at least three independent biological replicates (SH‐SY5Y, *n* = 5; KELLY, *n* = 4; BE(2)‐C, *n* = 3). (B) ATP13A3 siRNA‐mediated knockdown (KD) reduces ^3^H‐labeled putrescine (PUT) and spermidine (SPD) uptake in non‐*MYCN* amplified (SH‐SY5Y) and *MYCN*‐amplified (KELLY and BE(2)‐C) neuroblastoma cell lines. One sample *t*‐test was used to assess the significance of the decrease in uptake compared with scr‐ctrl cells. Graphs depict mean ± SEM of at least two independent experiments (SH‐SY5Y, *n* = 4; KELLY, *n* = 3; BE(2)‐C, *n* = 2). Statistical significance was defined as *P < 0.05, **P < 0.01, ***P < 0.001, ****P < 0.0001 or ns (non‐significant).

To further validate the role of ATP13A3 in polyamine uptake in various neuroblastoma cells, we performed transient siRNA‐mediated ATP13A3 knockdown in both *MYCN*‐amplified (KELLY and BE(2)‐C) and non‐*MYCN* amplified (SH‐SY5Y) neuroblastoma cell lines (Fig. 5A, Fig. S6). Compared with cells transfected with scrambled control siRNA (scr‐ctrl), cells with ATP13A3 knockdown presented a significantly reduced uptake of radiolabeled putrescine and spermidine (Fig. 5B), or BDP‐labeled polyamines (Fig. S7A). Similar effects were observed in SH‐SY5Y cells with miRNA‐mediated stable knockdown of ATP13A3 (Fig. S7B–C), while SLC3A2 and ATP13A2 expression were not significantly altered (Fig. S7D–F), consistent with our findings in the SH‐SY5Y ATP13A3 overexpression model (Fig. S4).

Combined, our experiments provide strong evidence for a key role of ATP13A3 in basal polyamine uptake in neuroblastoma cells.

### 3.4 ATP13A3 supports neuroblastoma cell growth

**Fig. 6:**
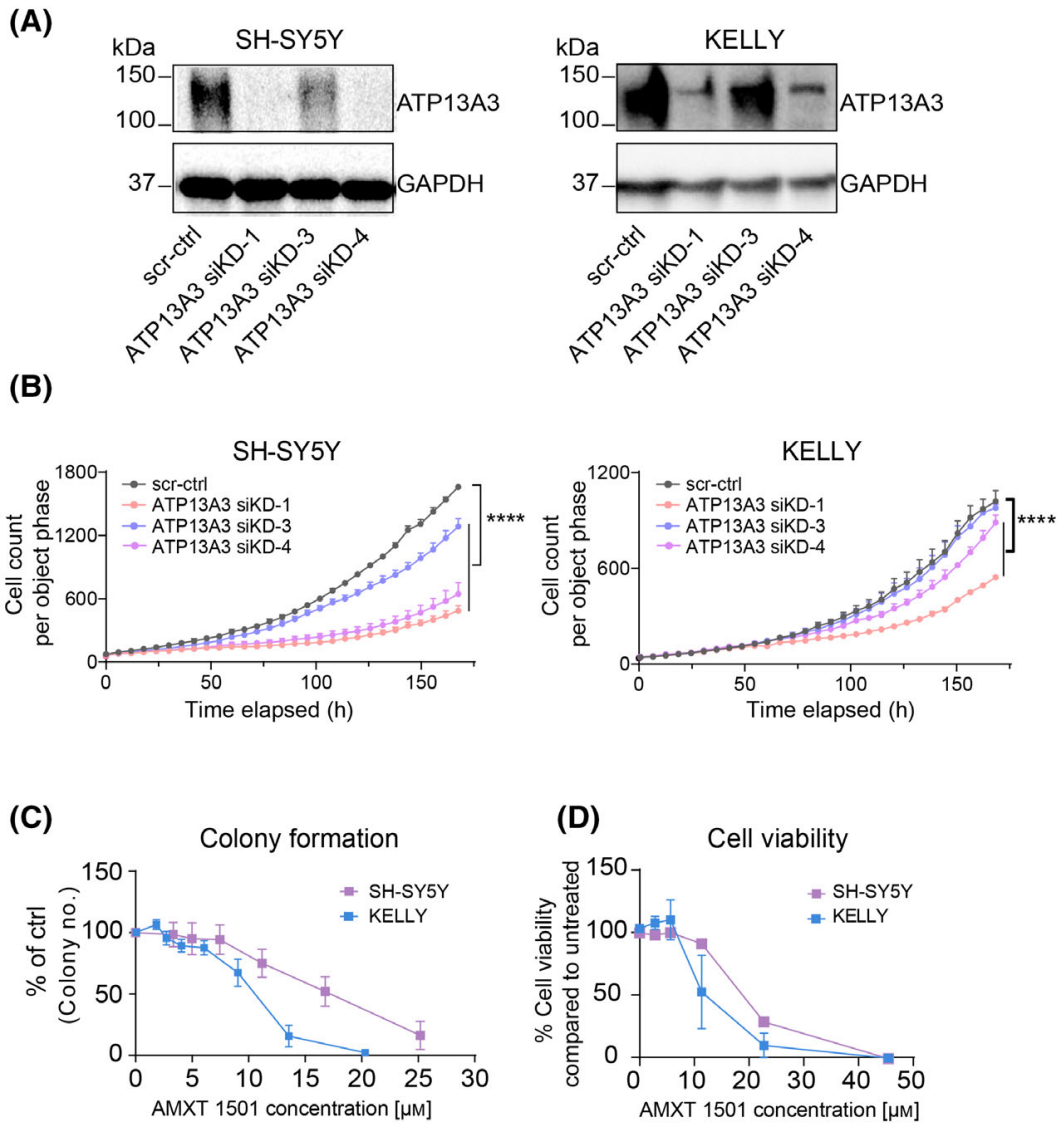
ATP13A3‐mediated polyamine uptake supports the growth of neuroblastoma cells. (A) Representative Western blots showing reduced ATP13A3 protein levels after siRNA‐mediated silencing of ATP13A3 in neuroblastoma cells (48 h). The depicted blot is representative for data obtained in at least two independently performed experiments. (B) Silencing of ATP13A3 decreases SH‐SY5Y and KELLY cell growth, as assessed by the IncuCyte live cell imaging system. Two‐way ANOVA was used for statistical analysis. Graphs depict mean ± SEM of three independent biological replicates. (C, D) AMXT 1501 treatment reduced neuroblastoma cell colony formation (C) and cell viability (after 72 h treatment) (D), in *MYCN*‐amplified (KELLY) and non‐*MYCN*‐amplified (SH‐SY5Y) neuroblastoma cell lines. Graphs depict mean ± SEM of three independent biological replicates. Statistical significance was defined as ****P < 0.0001 or ns (non‐significant).

Elevated *ATP13A3* expression in neuroblastoma patients is associated with worse survival, which suggests that ATP13A3‐mediated polyamine uptake may contribute to malignant neuroblastoma phenotypes, similar to ODC1‐mediated polyamine biosynthesis. We therefore assessed the impact of ATP13A3 silencing (Fig. 6A) on the growth and colony‐forming capacity of neuroblastoma cells. We observed that ATP13A3 silencing in both *MYCN*‐amplified (KELLY) and non‐*MYCN*‐amplified (SH‐SY5Y) neuroblastoma cells significantly attenuated cell growth and that this was a dose‐dependent effect as less effective silencing was associated with less pronounced effects on growth inhibition (Fig. 6B). These data indicate that targeting polyamine uptake via ATP13A3 impacts neuroblastoma cell growth, which is further confirmed by our observation that the polyamine uptake inhibitor AMXT 1501 [11, 26, 27], as a single agent, negatively impacted colony formation ability and viability of *MYCN*‐amplified KELLY and non‐*MYCN*‐amplified SH‐SY5Y neuroblastoma cells (Fig. 6C,D).

Hence, our data illustrate that neuroblastoma cells depend on ATP13A3‐mediated polyamine uptake to sustain their growth.

### 3.5 ATP13A3 mediates DFMO‐induced polyamine uptake in neuroblastoma cells

**Fig. 7:**
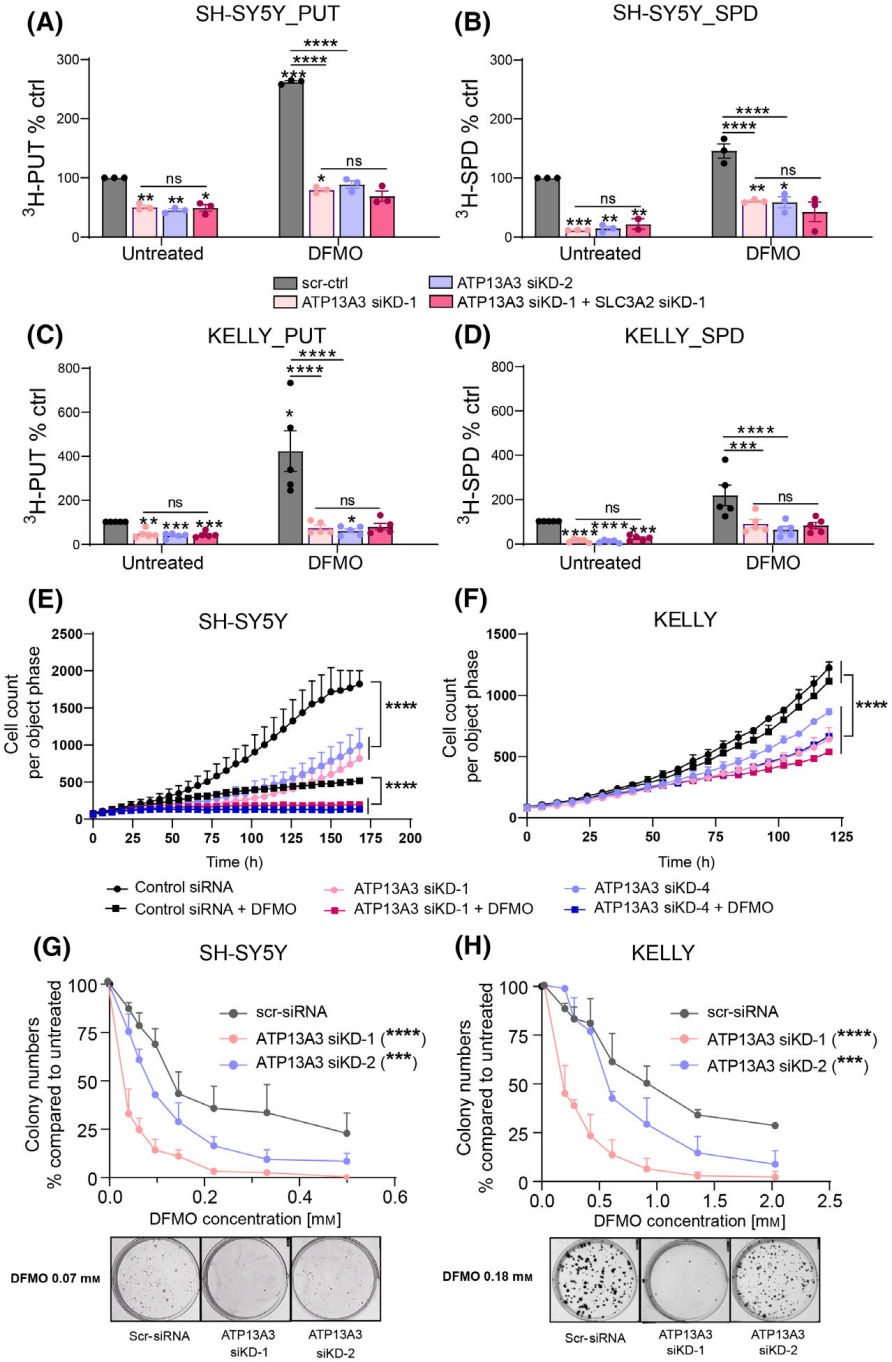
ATP13A3 silencing impairs DFMO‐induced polyamine uptake and increases DFMO sensitivity in neuroblastoma cells. (A–D) Radiolabeled putrescine (PUT) (A, C) and spermidine (SPD) (B, D) uptake in SH‐SY5Y (A, B) and KELLY (C, D) cell lines with or without difluoromethylornithine (DFMO) treatment, and in response to siRNA‐mediated silencing of ATP13A3 (ATP13A3 siRNA knockdown 1 and 2; ATP13A3 siKD‐1 and siKD‐2) alone or in combination with SLC3A2 siRNA knockdown (SLC3A2 siKD) *versus* scrambled siRNA control (scr‐ctrl). One sample *t*‐tests were used to compare uptake efficiency relative to untreated cells transfected with scr‐ctrl (asterisks on the bar). One‐way ANOVA was used for other group comparisons (asterisks between bars). Graphs depict mean ± SEM of at least three independent biological replicates (*n* = 3 for SH‐SY5Y PUT, *n* = 3 for SH‐SY5Y SPD except *n* = 2 for untreated ATP13A3 siKD‐1 + SLC3A2 siKD‐1; *n* = 5 for KELLY PUT and KELLY SPD). (E, F) Silencing of ATP13A3 reduced cell growth of SH‐SY5Y (E) and KELLY (F) cells upon treatment with DFMO as determined with the IncuCyte live cell imaging system. Two‐way ANOVA was used to compare growth curves. Graphs depict mean ± SEM of three independent biological replicates for SH‐SY5Y and two independent biological replicates for KELLY. (G, H) ATP13A3 knockdown sensitizes SH‐SY5Y (G) and KELLY (H) cells to DFMO treatment as measured by colony formation assays. Two‐way ANOVA was used to compare dose–response curves between groups. Graphs depict mean ± SEM of three independent replicates for SH‐SY5Y, two independent experiments for KELLY. Statistical significance was defined as *P < 0.05, **P < 0.01, ***P < 0.001, ****P < 0.0001 or ns (non‐significant).

Since the identity of the transporter(s) responsible for DFMO‐induced polyamine uptake remain(s) unknown, we next evaluated the consequences of silencing ATP13A3 on DFMO‐induced polyamine uptake. Knockdown of ATP13A3 alone was sufficient to prevent any DFMO‐induced compensatory increase in radiolabeled putrescine and spermidine uptake in both *MYCN*‐amplified and non‐*MYCN* amplified neuroblastoma cell lines (Fig. 7A–D, Fig. S8). Moreover, combined silencing of both ATP13A3 and SLC3A2 did not result in any further attenuation of uptake (Fig. 7A–D, Fig. S8). These results demonstrate that ATP13A3, but not SLC3A2, is a major contributor to the DFMO‐induced polyamine uptake. ATP13A3 silencing also sensitized *MYCN*‐amplified (KELLY) and non‐*MYCN*‐amplified (SH‐SY5Y) neuroblastoma cells to escalating DFMO concentrations, which resulted in reduced growth and colony‐forming capabilities (Fig. 7E–H).

Combined, our data demonstrate that ATP13A3 silencing counters the DFMO‐induced upregulation of polyamine uptake, and enhances the efficacy of DFMO, suggesting that ATP13A3 might be a clinically relevant therapeutic target to potentiate polyamine biosynthesis inhibition approaches in neuroblastoma.

### 3.6 The polyamine transport inhibitor AMXT 1501 inhibits ATP13A3‐mediated polyamine uptake in neuroblastoma cells

**Fig. 8:**
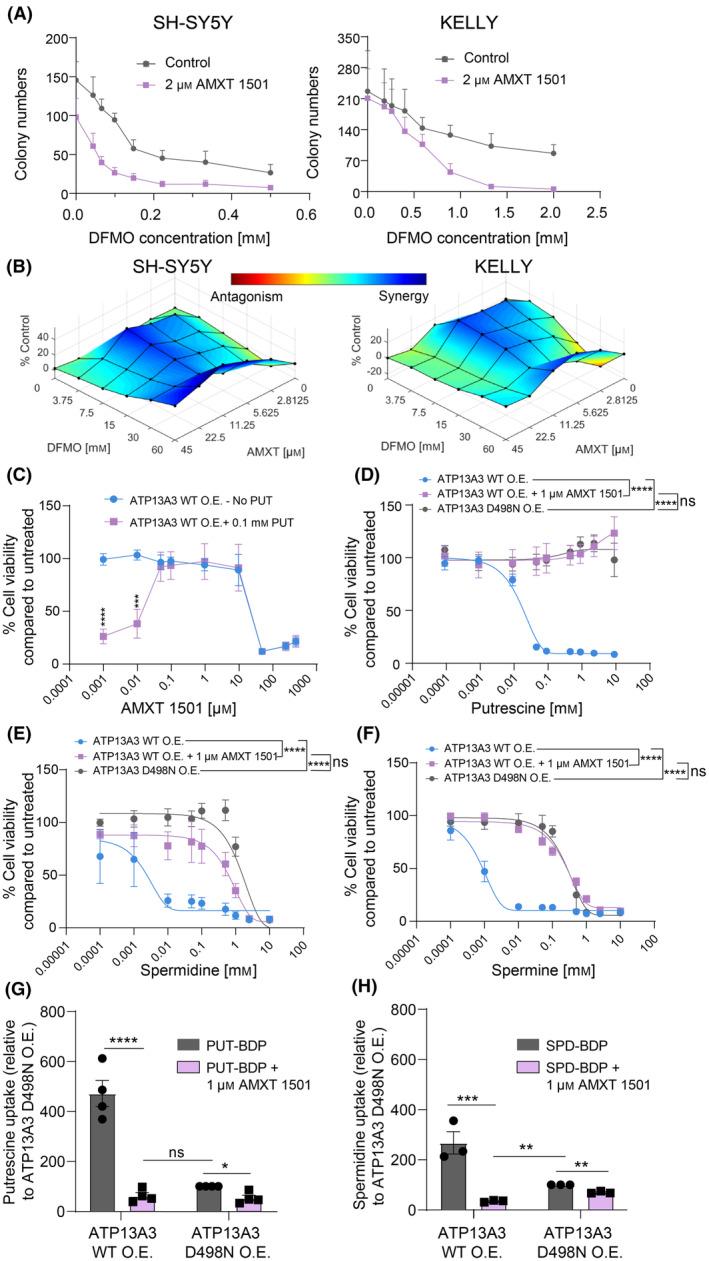
AMXT 1501 inhibits ATP13A3‐mediated polyamine uptake. (A) A fixed dose of AMXT 1501 enhanced the inhibitory effect of difluoromethylornithine (DFMO) on neuroblastoma colony formation of SH‐SY5Y and KELLY cells. Graphs depict mean ± SEM of three independent biological replicates. (B) SH‐SY5Y and KELLY cells were treated with increasing dose of DFMO and AMXT 1501 in a 6X6 format, and the synergy between these two drugs at different combination is visualized by Combenefit software, using Bliss synergy model, with blue color indicating strong synergy and red representing antagonism. The Synergy plot is generated from three independent biological replicates. (C) Cytotoxicity assay, using the 4‐methylumbelliferyl heptanoate (MUH) reagent to assess cell viability, showing the window of efficacy for AMXT 1501 in SH‐SY5Y cells overexpressing ATP13A3 wild type (WT O.E.). Graphs depict mean ± SEM of three independent biological replicates. Two‐way ANOVA was used to compare dose response curves of cells with or without PUT. (D–F) Overnight pre‐treatment of SH‐SY5Y cells overexpressing ATP13A3 WT with 1 μm AMXT 1501 protects them from toxic concentrations of putrescine (PUT) (D), spermidine (SPD) (E) and spermine (SPM) (F). Two‐way ANOVA was used to compare dose response curves in D‐F. Graphs depict mean ± SEM of three independent biological replicates. (G, H) Overnight pre‐treatment with 1 μm AMXT 1501 abolishes ATP13A3‐mediated PUT‐BDP (G) and SPD‐BDP (H) uptake in SH‐SY5Y cells overexpressing ATP13A3 WT as well as ATP13A3 D498N. One sample *t*‐test was used to compare mean uptake levels relative to untreated SH‐SY5Y cells overexpressing the ATP13A3 D498N. One‐way ANOVA was used to compare mean uptake levels for all other multiple comparisons. Graphs depict mean ± SEM of four independent biological replicates for PUT and three independent biological replicates for SPD. Experiments illustrated in figs E, F and H were performed in the presence of 1 mm aminoguanidine. Statistical significance was defined as *P < 0.05, **P < 0.01, ***P < 0.001, ****P < 0.0001 or ns (non‐significant).

The polyamine transport inhibitor AMXT 1501 blocks DFMO‐induced polyamine uptake [11, 12] and is currently being tested in combination with DFMO in clinical trials for solid cancers. We demonstrated earlier that AMXT 1501 as a single agent inhibits neuroblastoma cell viability and colony formation similar to ATP13A3 silencing (Fig. 6C,D). Since the exact target(s) of AMXT 1501 remain(s) unknown and ATP13A3 emerges as a likely target, we next examined whether AMXT 1501 inhibits ATP13A3‐mediated polyamine uptake. In line with our previous results and similar to ATP13A3 silencing [10, 12], AMXT 1501 effectively blocks putrescine and spermidine uptake and prevents the DFMO‐induced compensatory increase in polyamine uptake in both *MYCN*‐amplified and non‐*MYCN* amplified neuroblastoma cells (Fig. S9). Reminiscent of ATP13A3 silencing, a sublethal dose of AMXT 1501 also sensitized *MYCN*‐amplified and non‐*MYCN*‐amplified neuroblastoma cells to DFMO in colony formation assays (Fig. 8A). Formal 6 × 6 matrix synergy assays demonstrated strong synergy between DFMO and AMXT 1501 as determined based on the Bliss Independence model (Fig. 8B) [22].

To provide more conclusive evidence for AMXT 1501 inhibiting ATP13A3‐mediated polyamine uptake in neuroblastoma, we next assessed whether the activity of ATP13A3 can be effectively hindered by AMXT 1501. We determined the uptake inhibition capacity of AMXT 1501 by evaluating the potential of AMXT 1501 to abrogate the cytotoxic effects of putrescine supplementation in cells overexpressing ATP13A3 (Fig. 8C). We defined the concentration of AMXT 1501 required to prevent 50% of putrescine toxicity by blocking ATP13A3‐mediated polyamine import, *that is*, the IC_50_ for polyamine transport inhibition, to be approximately 0.05 μm. Additionally, AMXT 1501 by itself exerted cytotoxic activity with IC_50_ value around 50 μm. As AMXT 1501 is a polyamine analog, we repeated these findings in the presence of 1 mm aminoguanidine to account for serum amine oxidases present in serum added to culture medium. In the presence of 1 mm aminoguanidine, AMXT 1501 presented a higher potency to abrogate putrescine‐induced cytotoxicity an IC_50_ of approximately 0.01 μm (Fig. S10). The increase in AMXT 1501's potency in the presence of 1 mm aminoguanidine suggests that AMXT 1501 may be subjected to breakdown through serum oxidases. However, the toxicity of AMXT 1501 itself seen at higher doses remained unchanged even with the addition of aminoguanidine (Fig. S10). These observations suggest that the mechanism behind AMXT 1501's intrinsic toxicity is independent of the toxic by‐products generated by its breakdown by serum oxidases. Moreover, aminoguanidine addition did not affect AMXT 1501's inhibitory action on colony formation capacity of ATP13A3 WT and ATP13A3 D498N expressing SH‐SY5Y cells (Fig. S11). Based on these findings, we proceeded to pretreat ATP13A3 overexpressing SH‐SY5Y cells with a non‐toxic concentration of 1 μm AMXT 1501 that is able to block polyamine transport of putrescine. AMXT 1501 rescued the toxicity induced by putrescine, spermidine and spermine in SH‐SY5Y cells overexpressing ATP13A3 WT to the point that their viability was comparable to that of SH‐SY5Y cells overexpressing ATP13A3 D498N (Fig. 8D–F). Similarly, AMXT 1501 treatment completely inhibited ATP13A3‐mediated putrescine‐BDP and spermidine‐BDP uptake (Fig. 8G,H). Viability and uptake experiments were performed in the presence of 1 mm aminoguanidine, except with putrescine or putrescine‐BDP (Fig. 8C,D,G). Indeed, putrescine is not degraded by serum oxidases [19, 28], and aminoguanidine addition had no effect on the relative uptake window of putrescine‐BDP uptake or cytotoxicity in ATP13A3 WT versus D498N expressing cells (Fig. S5A,D). Aminoguanidine only rescued ATP13A3 D498N, but not ATP13A3 WT expressing cells from spermidine and spermine toxicity. The toxicity in ATP13A3 D498N expressing cells, occurring at higher extracellular polyamine levels, thus most likely results from extracellular serum oxidases. In contrast, ATP13A3 WT expressing cells suffer from intracellular polyamine overload following polyamine uptake, which aminoguanidine cannot prevent.

Our data thus provide compelling evidence that AMXT 1501 effectively targets ATP13A3‐mediated polyamine uptake in neuroblastoma cells, underscoring the therapeutic promise of ATP13A3 targeting in neuroblastoma.

### 3.7 *ATP13A3* is a MYC target gene

**Fig. 9:**
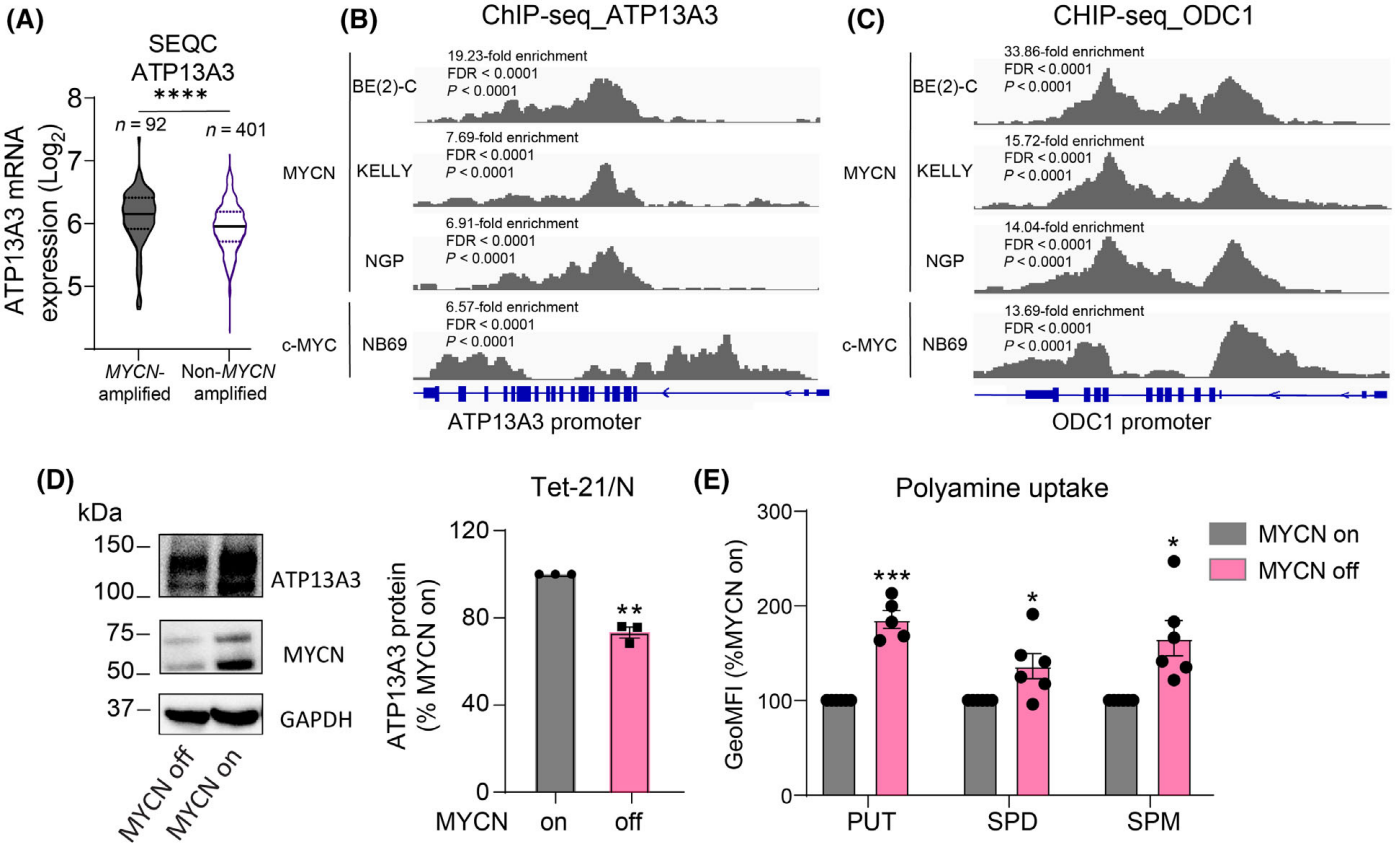
MYCN expression and polyamine uptake in neuroblastoma cells. (A) mRNA expression (Transcripts Per Million, TPM) of polyamine transporters in the SEQC neuroblastoma database (*n* = 498; graph shows only *n* = 493 as 5 patients have unknown *MYCN* status). Graph represents the distribution of data density of ATP13A3 expression in neuroblastoma patients, with the central line representing the median, and the upper and lower lines indicating the third (Q3) and first (Q1) quartiles. Student's *t*‐test was used for statistical analysis. (B, C) ChIP‐seq tracks of *ATP13A3* (B) and *ODC1* (C) after pulldown with anti‐MYCN antibody in three *MYCN*‐amplified neuroblastoma cell lines, BE(2)‐C, KELLY, and NGP, and with anti‐c‐MYC antibody in NB69 neuroblastoma cells, with one replicate in each cell line. Statistical analysis of ChIP‐seq data was performed using MACS2 as described in method section. (D) Western blot assessing ATP13A3 and MYCN expression after 72 h of doxycycline treatment. Representative blots from three independent experiments. Graphs depict mean ± SEM of three independent replicates. One sample *t*‐test was used to assess the significance of the difference between ATP13A3 protein levels of MYCN^−^ cells and that of MYCN^+^ cells (100%) which is indicated by the asterisks on top of the bars. (E) Uptake of BODIPY (BDP)‐labeled polyamines (putrescine, PUT; spermidine, SPD; spermine, SPM) in Tet‐21/N cells with or without MYCN expression. One sample *t*‐test was used to compare uptake levels, measured by geometric mean fluorescence intensities (GeoMFI), relative to MYCN^+^ cells. Graphs depict mean ± SEM of at least five independent biological replicates (*n* = 5 for PUT; *n* = 6 for SPD and SPM). Statistical significance was defined as *P < 0.05, **P < 0.01, ***P < 0.001, ****P < 0.0001 or ns (non‐significant).

Our findings support a role for ATP13A3‐mediated polyamine uptake in both *MYCN*‐amplified and non‐*MYCN* amplified neuroblastoma cells. Interestingly, we observed that, as previously found for *SLC3A2* [10], *ATP13A3* expression was significantly higher in *MYCN*‐amplified *versus* non‐*MYCN* amplified neuroblastoma tumors (Fig. 9A). As *MYCN* gene amplification is the strongest genetic predictor for poor prognosis in neuroblastoma and has been shown to drive polyamine synthesis and uptake with *ODC1* and *SLC3A2* being identified as *bona fide* MYCN targets [10, 29, 30], we assessed whether the observed association between high *ATP13A3* expression and poor outcome may be mediated by *MYCN* amplification. Multivariate survival analyses on two neuroblastoma cohorts (Kocak GSE45547; SEQC GSE62564) that incorporated *MYCN* amplification, patient age and tumor stage as other prognostic variables, indicated that high *ATP13A3* expression is an independent prognostic marker for neuroblastoma outcome (Table 1). We subsequently assessed whether *ATP13A3* is a target gene of the family of MYC transcription factors, such as *ODC1* and *SLC3A2*. We analyzed publicly available ChIP‐seq databases and found that similar to *ODC1*, MYCN binds to the promoter of *ATP13A3*, although the fold enrichment at the *ODC1* promoter is much higher than at the *ATP13A3* promoter (Fig. 9B,C). Furthermore, c‐MYC also binds the *ATP13A3* and *ODC1* promoter in neuroblastoma cells, albeit at a different region than MYCN, potentially denoting differences in MYCN and c‐MYC‐mediated regulation of expression (Fig. 9B,C). Our analysis suggests that *ATP13A3* is a MYC target gene like *ODC1* and *SLC3A2* and that high expression of *ATP13A3* represents an independent prognostic predictor of poor outcome in neuroblastoma.

**Table 1:** Multivariate survival analysis in two neuroblastoma patient cohorts. Cohorts used: (i) Kocak GSE45547 (649 patients) and (ii) SEQC GSE62564 (498 patients). Cox proportional hazards analysis adjusted for *MYCN* status (amplified vs. non‐amplified), stage (favorable vs. unfavorable) and age at diagnosis (< 18 months vs. > 18 months). Numbers indicated in bold are hazard risks of high *ATP13A3* expression with patients dichotomized around the upper quartile (UQ). Hazard risks > 1 indicate unfavorable contribution to patient survival.

		Multivariate analysis			
		Event‐free survival		Overall survival	
		Relative Hazard (95% CI)	*P*	Relative Hazard (95% CI)	*P*
Kocak	ATP13A3 high expression	1.28 (0.96–1.72)	0.0955	1.58 (1.12–2.24)	0.00926
	Unfavorable (stage 3/4)	2.11 (1.54–2.89)	3.72E‐06	4.15 (2.61–6.60)	1.76E‐09
	Older age (≥ 18 months)	2.40 (1.69–3.41)	9.90E‐07	4.85 (2.62–8.97)	5.06E‐07
	MYCN amplification	1.70 (1.23–2.35)	0.00129	2.91 (2.04–4.16)	4.62E‐09
SEQC	ATP13A3 high expression	1.47 (1.06–2.03)	0.0223	1.47 (1.06–2.03)	0.0708
	Older age (≥ 18 months)	0.83 (0.46–1.51)	0.5455	5.51 (0.76–39.87)	0.0911
	MYCN amplification	2.88 (2.05–4.05)	1.19E‐09	6.31 (4.14–9.63)	<2e‐16

It is well‐established that ODC1‐mediated polyamine biosynthesis is one of the mechanisms by which *MYCN* amplification drives neuroblastoma tumor cell proliferation [30]. We next evaluated whether MYCN also drives polyamine uptake, as has previously been suggested for pancreatic adenocarcinoma [31], by utilizing Tet‐21/N neuroblastoma cells with doxycycline‐inducible MYCN silencing [10]. In line with our database and ChIPseq analyses, ODC1 and ATP13A3 expression decreased following MYCN silencing (Fig. 9D, Fig. S12), but surprisingly, a higher basal uptake of BDP‐labeled polyamines was observed both in the presence or absence of aminoguanidine (Fig. 9E, Fig. S13). Since MYCN may have a stronger impact on the transcriptional regulation of *ODC1* than of *ATP13A3*, neuroblastoma cells may be more dependent on biosynthesis and less dependent on polyamine uptake when MYCN is expressed.

**Fig. 10:**
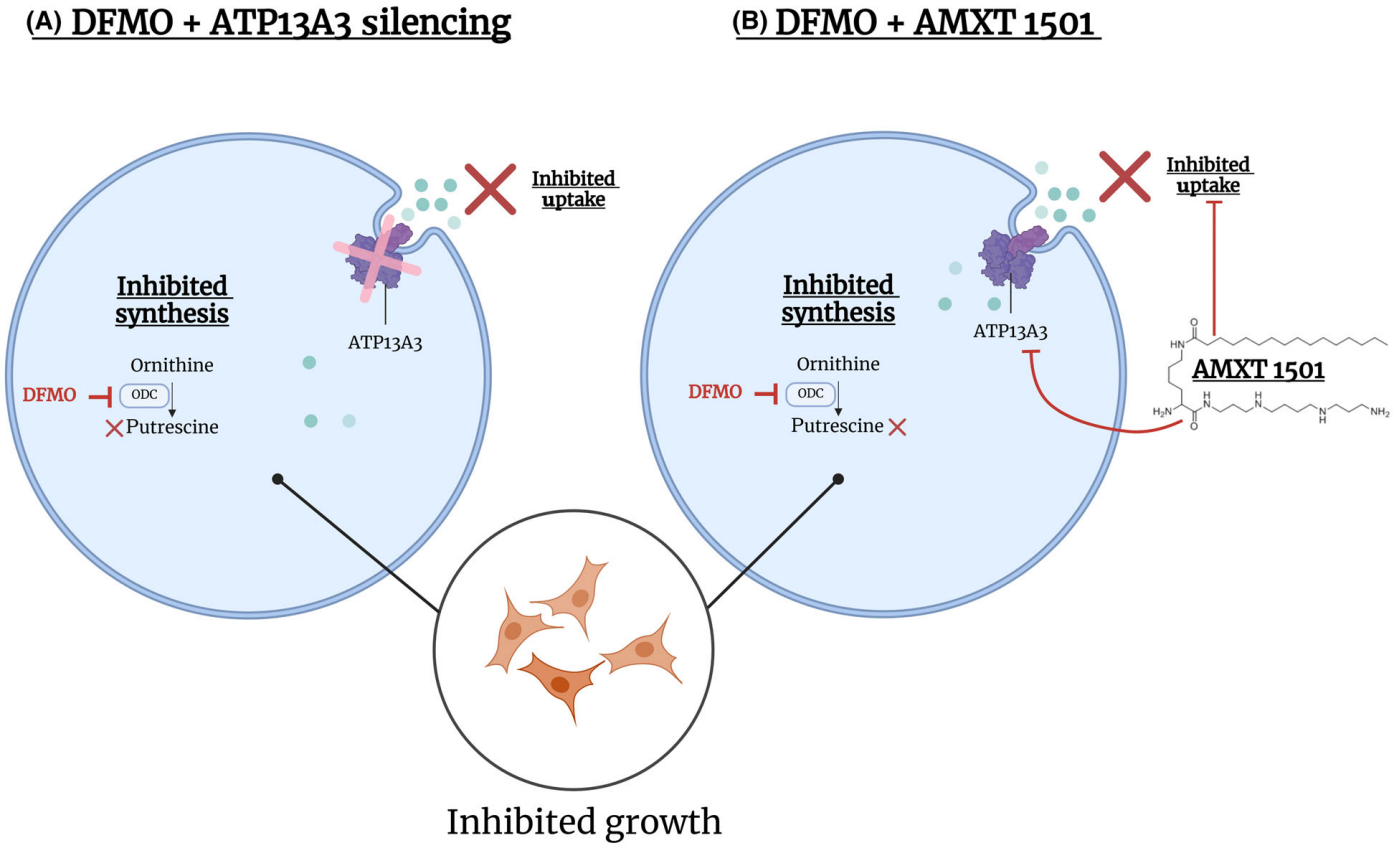
ATP13A3 as a novel therapeutic target for neuroblastoma. Targeting ATP13A3 either by (A) its silencing or (B) with AMXT 1501, in conjunction with difluoromethylornithine (DFMO), prevents DFMO‐induced compensatory polyamine uptake and inhibits the growth of neuroblastoma cells.

In conclusion, this study identified ATP13A3 as a critical regulator of basal and DFMO‐induced polyamine uptake in *MYCN*‐amplified and non‐*MYCN*‐amplified neuroblastoma cells, which is targetable by the polyamine transport inhibitor AMXT 1501, and thereby highlights ATP13A3 as a novel therapeutic target for neuroblastoma (Fig. 10).

## 4 Discussion

### 4.1 ATP13A3 is a major polyamine transporter in neuroblastoma

Neuroblastoma cells are addicted to polyamines for their growth and survival and these cells respond to inhibition of polyamine biosynthesis by DFMO via upregulating polyamine uptake as a rescue mechanism to prevent polyamine depletion [24, 25]. In this study we explored two candidate polyamine transporters, SLC3A2 and ATP13A3, as their higher expression is associated with an inferior outcome in neuroblastoma patients. We, and others, have previously highlighted ATP13A3 as a mammalian polyamine transporter [14, 16, 31, 32]. Although there is no biochemical evidence yet on purified SLC3A2 for its polyamine transporter function, in a cellular context, SLC3A2 has previously been implicated in both the export and uptake of polyamines as well as their acetylated forms [15]. Moreover, aside from the transport of polyamines to and from the extracellular environment, SLC3A2 has been demonstrated to affect polyamine homeostasis as a whole. This could in part be due to the fact that SLC3A2 also mediates the cellular uptake of arginine [33] which is both a precursor to ornithine for polyamine synthesis as well as for nitric oxide which may regulate polyamine uptake [15, 34]. In investigating and comparing the potential roles of SLC3A2 and ATP13A3 in polyamine uptake under basal and DFMO‐treated conditions, our work highlights notable similarities and differences in the role of both candidate transporters in neuroblastoma. We demonstrated increased intracellular polyamine levels as well as an enhanced uptake of exogenous polyamines when ATP13A3 was overexpressed in neuroblastoma cells. In contrast, SLC3A2 overexpression did not enhance polyamine uptake in neuroblastoma cells. Similarly, while the silencing of either ATP13A3 or SLC3A2 in neuroblastoma cells resulted in reduced polyamine uptake, only the silencing of ATP13A3, and not of SLC3A2, was sufficient to prevent the compensatory increase in polyamine uptake under DFMO treatment.

Although it is unclear why silencing SLC3A2 decreases polyamine uptake while its overexpression does not increase it, it needs to be considered that SLC3A2 fulfills its multifaceted roles by acting as a “hub protein” directly interacting with numerous binding partners [35] so that overexpression of SLC3A2 alone, without other co‐factors, may not be sufficient to induce an increase in polyamine uptake.

Nevertheless, in a direct side‐by‐side comparison, ATP13A3 has a stronger impact on polyamine uptake than SLC3A2 in neuroblastoma cells under basal and DFMO‐treatment conditions. Thus, our study positions ATP13A3 as a primary polyamine transporter responsible for polyamine uptake in neuroblastoma.

### 4.2 ATP13A3 emerges as a new therapeutic target for neuroblastoma and other cancers

In cells, ATP13A3‐mediated polyamine uptake can be prevented by AMXT 1501, a polyamine uptake inhibitor currently in phase I/II clinical trials for cancer, indicating that preventing polyamine uptake via ATP13A3 may at least partially explain the mode of action of the drug. AMXT 1501 also inhibited neuroblastoma cell growth and colony formation showing that polyamine uptake in basal conditions is important for neuroblastoma growth. The growth and colony formation capacity of neuroblastoma cells were also reduced upon ATP13A3 knockdown, which is consistent with high ATP13A3 expression being predictive of poor outcome in neuroblastoma patients. Additionally, ATP13A3 knockdown increased the sensitivity of neuroblastoma cells to DFMO pointing to synergistic effects of blocking polyamine uptake via ATP13A3 and inhibiting polyamine synthesis with DFMO. These results indicate that inhibition of ATP13A3, possibly in combination with DFMO, may be of therapeutic interest for neuroblastoma. Based on our findings, ATP13A3 emerges as a major polyamine transporter in neuroblastoma that represents a target of the drug AMXT 1501.

Studies in other cancers including pancreatic, head, neck, and cervical cancers, have noted associations between ATP13A3 expression and poorer overall survival, suggesting a role for this polyamine uptake pathway in other cancer types as well, although detailed studies into the role of this transporter in most cancers are largely lacking [16, 31, 36, 37, 38]. ATP13A3 was highlighted as the major importer of spermidine and spermine in human pancreatic cells, and metastatic pancreatic cancer cells displayed higher polyamine import and expression of ATP13A3 as compared to cells with slow proliferation. ATP13A3 knockdown significantly decreased the growth of human pancreatic cancer cells under DFMO treatment [31]. Also, other members of the P5B‐ATPase family have been linked to different cancers [15, 17]. ATP13A2 has been implicated in melanoma, colon cancer, hepatocellular carcinoma, acute myeloid leukemia and non‐small‐cell lung cancer [39, 40, 41, 42, 43]. We also reported that high ATP13A4 expression explains the increased polyamine uptake in the breast cancer cell line MCF7 compared to the non‐tumorigenic epithelial breast cell line MCF10A [17]. ATP13A4 amplification has been frequently observed in patients with non‐small‐cell lung cancer ovarian cancer, cervical cancer, head and neck cancer, endometrial cancer, uterine endometrioid carcinoma, bladder cancer, melanoma, and breast cancer [17].

### 4.3 Toward P5B‐type ATPase inhibitors for cancer therapy

The discovery of ATP13A3 as the key driver in the hyperactive polyamine transport system of neuroblastoma may pave the way for developing more targeted strategies to attack cancer cells that heavily rely on increased polyamine import. To deprive neuroblastoma cells of their polyamine addiction, selective ATP13A3 inhibitors can be developed. This is of particular importance as AMXT 1501, being a polyamine analog, may also target other polyamine transporters alongside ATP13A3, and may be subjected to polyamine competition in the tumor environment. Our database analyses revealed that ATP13A2 is expressed to similar levels as ATP13A3 in neuroblastoma patients and, contrary to ATP13A3 and SLC3A2 expression, high expression of ATP13A2 is predictive of a better outcome in patients. Indeed ATP13A2, being a lysosomal polyamine exporter, has a protective role in lysosomes; and its loss of function has been implicated in a wide variety of neurodegenerative diseases [13, 15, 44]. Although the safety of AMXT 1501 (in combination with DFMO) has been established in phase I/II clinical trials (NCT03536728; NCT05500508), targeting ATP13A3 with specific inhibitors also may not be without risk, since *ATP13A3* gene mutations have been associated with the pathogenicity of pulmonary arterial hypertension [32, 45]. Future analysis of conditional *Atp13a3* knockout mice may help establish the (patho)physiological consequences of long‐term *versus* short‐term *Atp13a3* deficiency.

### 4.4 ATP13A3 as a potential biomarker or therapeutic target under DFMO treatment

DFMO is currently under investigation in a clinical trial in children with neuroblastoma in combination with standard of care chemotherapy and dinutuximab (NCT05500508). Studies by us and others unequivocally demonstrated that cancer cells in general, and neuroblastoma cells in particular, compensate for inhibition of polyamine synthesis by boosting uptake of polyamines from the extracellular environment [24, 25]. The compensatory polyamine uptake phenomenon can be explained by the tight regulation of intracellular polyamine levels by the interplay between antizyme and antizyme‐inhibitor. Antizyme inhibits both polyamine synthesis and polyamine uptake, and is regulated by intracellular polyamine levels [46]. This mechanism is mediated by Antizyme Inhibitor, the expression of which increases after polyamine depletion upon DFMO treatment, resulting in increased polyamine uptake [47, 48, 49]. Here, we show a role for ATP13A3 in the DFMO‐induced polyamine uptake in neuroblastoma cells suggesting that ATP13A3 may contribute to the resistance mechanisms employed by neuroblastoma cells following DFMO treatment. Therefore, combination therapies with DFMO and ATP13A3 inhibitors may be of therapeutic interest to prevent this compensatory response. Moreover, information regarding ATP13A3 expression or activity may help delineate which patients are more likely to develop rescue mechanisms to polyamine biosynthesis inhibition treatments with DFMO.

While it remains unclear whether ATP13A3 levels can be used to predict responsiveness to DFMO, we noted a trend toward an increase in ATP13A3 protein levels in response to DFMO treatment of neuroblastoma cells (data not shown). In pancreatic cancer cells however, the expression level of ATP13A3 is inversely correlated with their sensitivity to polyamine transport inhibitors. To inhibit growth, pancreatic cancer cells with higher ATP13A3 expression required higher concentrations of polyamine transport inhibitors but lower concentrations of DFMO [31]. Conversely, in this study in neuroblastoma, we observed in neuroblastoma cells that ATP13A3 knockdown increases sensitivity to DFMO, suggesting that high ATP13A3 expression might drive resistance to DFMO. These contradictory findings in neuroblastoma *versus* pancreatic cancer cells highlight the importance to thoroughly assess the functions of polyamine transporters in different cancer types.

To further validate ATP13A3 as a therapeutic target and assess its biomarker value in predicting responsiveness to DFMO in neuroblastoma, *in vivo* xenograft studies will be required to determine the efficacy of DFMO, AMXT 1501 and their combination in a panel of patient‐derived xenograft models with varying expression levels of ATP13A3 and/or ODC1.

### 4.5 ATP13A3 is a target of the *MYC* oncogene

The mechanisms regulating ATP13A3 expression are poorly understood. Our analysis of ChIP‐seq databases of *MYCN*‐amplified and c‐MYC‐expressing neuroblastoma cells, suggests that both MYCN and c‐MYC can directly bind the promotor of *ATP13A3*, though each at different regions, and thus may regulate *ATP13A3* mRNA levels. Importantly, MYC transcription factor binding properties are influenced by binding affinities to different consensus sequences as well as the binding of other interactors, which further adds to the complexity of *ATP13A3* and *ODC1* gene expression in different cell contexts [50]. Supporting this finding, *ATP13A3* mRNA levels were significantly higher in *MYCN*‐amplified *versus* non‐*MYCN*‐amplified neuroblastomas. The MYCN‐polyamine axis is of particular significance in neuroblastoma research, since multiple genes within the polyamine pathway, including *ODC1* and *SLC3A2*, are direct targets of MYCN [30]. The link between MYCN and the polyamine pathway is so well established that targeting the polyamine homeostasis pathway is seen as an indirect method of targeting MYCN which has been deemed undruggable [51, 52]. In relation to ATP13A3 however, high *ATP13A3* expression levels negatively correlate with survival of neuroblastoma patients, irrespective of *MYCN* status. Moreover, multivariate analysis showed that elevated *ATP13A3* expression is an independent predictor of poor prognosis in neuroblastoma, independent of *MYCN* amplification. Also, ATP13A3 knockdown appears effective in inhibiting growth in both *MYCN*‐amplified and non‐*MYCN* amplified neuroblastoma cell lines. While our ChIP‐seq database analyses point toward c‐MYC/MYCN directly regulating ATP13A3 expression through binding to the gene's promotor, dedicated promotor‐binding studies as previously performed for other polyamine pathway genes are required to provide firm evidence for this premise [10]. Further establishing the link between c‐MYC/MYCN and ATP13A3 would be very promising as such observations have been reported in pancreatic cancer cells where the correlation between expression levels of c‐MYC, ATP13A3 and polyamine uptake appear to predict the cells' responsiveness to polyamine depletion therapy (*i.e*., DFMO + a polyamine transport inhibitor such as AMXT 1501) [31].

## 5 Conclusion

Our data show that ATP13A3 represents an interesting novel therapeutic target to inhibit the polyamine transport system in neuroblastoma to increase the efficacy of DFMO treatment and limit neuroblastoma growth.

## Conflict of interest

MRB is the Founder, President and CSO at Aminex Therapeutics where he is an employee and stock owner. PV is involved in polyamine transporter drug screening efforts for cancer and Parkinson's disease.

## Author contributions

MA and WG are shared first authors and MH, PV, and KS are shared last and co‐corresponding authors. WG, MA, LX, PV, and KS conceptualized the study with contributions of RP, MDN, and MH. The stable cell line models were designed and generated by CVdH. SV synthesized and provided the BODIPY‐labeled polyamines. MRB supplied AMXT 1501 and contributed to interpretation of generated data. All experiments were performed by WG and MA, with support by MK, AK, DS, ER, AB, YF, and AS. XG and CM performed multivariate survival and ChIPseq database analyses. WG and MA analyzed the data. MA and WG wrote the first draft of the manuscript, which has been revised and edited by PV and KS. All authors have read and approved the manuscript.

### Peer review

The peer review history for this article is available at https://www.webofscience.com/api/gateway/wos/peer‐review/10.1002/1878‐0261.13789.

## Supporting information


**Fig. S1.** High expression of ATP13A3 is associated with significantly worse outcome in neuroblastoma patients.
**Fig. S2.** High expression of *ATP13A2* is associated with a better prognosis in neuroblastoma patients.
**Fig. S3.** Protein expression of SLC3A2 in SH‐SY5Y and KELLY cells at several time points after siRNA‐mediated silencing with four different siRNAs.
**Fig. S4.** qPCR analysis showing that *SLC3A2* or *ATP13A2* expression does not change upon the overexpression of ATP13A3 WT or D498N (dead mutant) in SH‐SY5Y cells.
**Fig. S5.** Effect of amino guanidine addition on polyamine uptake and toxicity.
**Fig. S6.** siRNA‐mediated ATP13A3 silencing decreased ATP13A3 protein expression levels in KELLY and SH‐SY5Y cells from 24 h after transfection for three (siKD‐1, siKD‐2, siKD‐4) out of four siRNAs.
**Fig. S7.** Impact of ATP13A3 silencing on polyamine uptake and polyamine transporter expression in neuroblastoma cells.
**Fig. S8.** Western blots confirming siRNA‐mediated knockdown of either ATP13A3, SLC3A2, or both compared with scrambled siRNA controls (scr‐ctrl) in SH‐SY5Y and KELLY cell lines.
**Fig. S9.** Overnight pretreatment with 1 μM AMXT 1501 inhibits baseline and DFMO‐induced polyamine uptake in neuroblastoma cells.
**Fig. S10.** Cytotoxicity assay, using the MUH reagent to assess cell viability, showing the window of efficacy for AMXT 1501 in SH‐SY5Y cells overexpressing ATP13A3 WT in the presence of 1 mM aminoguanidine.
**Fig. S11.** The addition of 1 mM aminoguanidine (AG) does not affect the inhibitory effect of AMXT 1501 on colony formation of SH‐SY5Y cells overexpression ATP13A3.
**Fig. S12.** MYCN silencing decreases *ODC1* expression in Tet‐21/N cells.
**Fig. S13.** Uptake of BODIPY‐labeled polyamines in Tet‐21/N cells with or without MYCN expression in the presence and absence of 1 mM aminoguanidine.
**Table S1.** Metabolomics data displayed as absolute metabolite concentrations in SH‐SY5Y cells overexpressing ATP13A3 WT or the catalytically dead ATP13A3 D498N mutant.

## Data Availability

The raw data generated in this study are deposited at Zenodo (DOI: 10.5281/zenodo.11652529). Data can also be provided upon request from the corresponding authors.
