# Correction to “The polyamine transporter ATP13A3 mediates difluoromethylornithine‐induced polyamine uptake in neuroblastoma”

**DOI:** 10.1002/1878-0261.70088

**Published:** 2025-07-02

Azfar M, Gao W, Van den Haute C, Xiao L, Karsa M, Pandher R, Karsa A, Spurling D, Ronca E, Bongers A, Guo X, Mayoh C, Fayt Y, Schoofs A, Burns MR, Verhelst SHL, Norris MD, Haber M, Vangheluwe P, Somers K. The polyamine transporter ATP13A3 mediates difluoromethylornithine‐induced polyamine uptake in neuroblastoma. *Mol Oncol.* 2025; 19: 913–936. https://doi.org/10.1002/1878‐0261.13789 Epub 2025 Feb 21. PMID: 39981745; PMCID: PMC11887671.

In the manuscript by Azfar, Mujahid et al., a typographical error in the acknowledgments section of the paper has been noted. The grant number for the Fonds voor Wetenschappelijk Onderzoek (FWO) PhD fellowship is 1SD6125N instead of 1S26125N. The authors acknowledge an oversight that occurred on their part and have revised the Acknowledgment section to mention the right grant number.

The correction of these errors has not altered the interpretation of the data and has not impacted the conclusions presented in the article. The authors have checked the entire document and agree to this correction.

We apologize for this error.

