# Supplementary material for: The polyamine transporter ATP13A3 mediates difluoromethylornithine‐induced polyamine uptake in neuroblastoma
Source: Mol Oncol. 2025 Feb 21;19(3):913–36. doi: 10.1002/1878-0261.13789 (PMC11887671; doi:10.1002/1878-0261.13789)
Supplement: Supplementary file 1 — Fig. S1. High expression of ATP13A3 is associated with significantly worse outcome in neuroblastoma patients. Fig. S2. High expression of ATP13A2 is associated with a better prognosis in neuroblastoma patients. Fig. S3. Protein expression of SLC3A2 in SH‐SY5Y and KELLY cells at several time points after siRNA‐mediated silencing with four different siRNAs. Fig. S4. qPCR analysis showing that SLC3A2 or ATP13A2 expression does not change upon the overexpression of ATP13A3 WT or D498N (dead mutant) in SH‐SY5Y cells. Fig. S5. Effect of amino guanidine addition on polyamine uptake and toxicity. Fig. S6. siRNA‐mediated ATP13A3 silencing decreased ATP13A3 protein expression levels in KELLY and SH‐SY5Y cells from 24 h after transfection for three (siKD‐1, siKD‐2, siKD‐4) out of four siRNAs. Fig. S7. Impact of ATP13A3 silencing on polyamine uptake and polyamine transporter expression in neuroblastoma cells. Fig. S8. Western blots confirming siRNA‐mediated knockdown of either ATP13A3, SLC3A2, or both compared with scrambled siRNA controls (scr‐ctrl) in SH‐SY5Y and KELLY cell lines. Fig. S9. Overnight pretreatment with 1 μM AMXT 1501 inhibits baseline and DFMO‐induced polyamine uptake in neuroblastoma cells. Fig. S10. Cytotoxicity assay, using the MUH reagent to assess cell viability, showing the window of efficacy for AMXT 1501 in SH‐SY5Y cells overexpressing ATP13A3 WT in the presence of 1 mM aminoguanidine. Fig. S11. The addition of 1 mM aminoguanidine (AG) does not affect the inhibitory effect of AMXT 1501 on colony formation of SH‐SY5Y cells overexpression ATP13A3. Fig. S12. MYCN silencing decreases ODC1 expression in Tet‐21/N cells. Fig. S13. Uptake of BODIPY‐labeled polyamines in Tet‐21/N cells with or without MYCN expression in the presence and absence of 1 mM aminoguanidine. Table S1. Metabolomics data displayed as absolute metabolite concentrations in SH‐SY5Y cells overexpressing ATP13A3 WT or the catalytically dead ATP13A3 D498N mutant. [file MOL2-19-913-s001.docx]

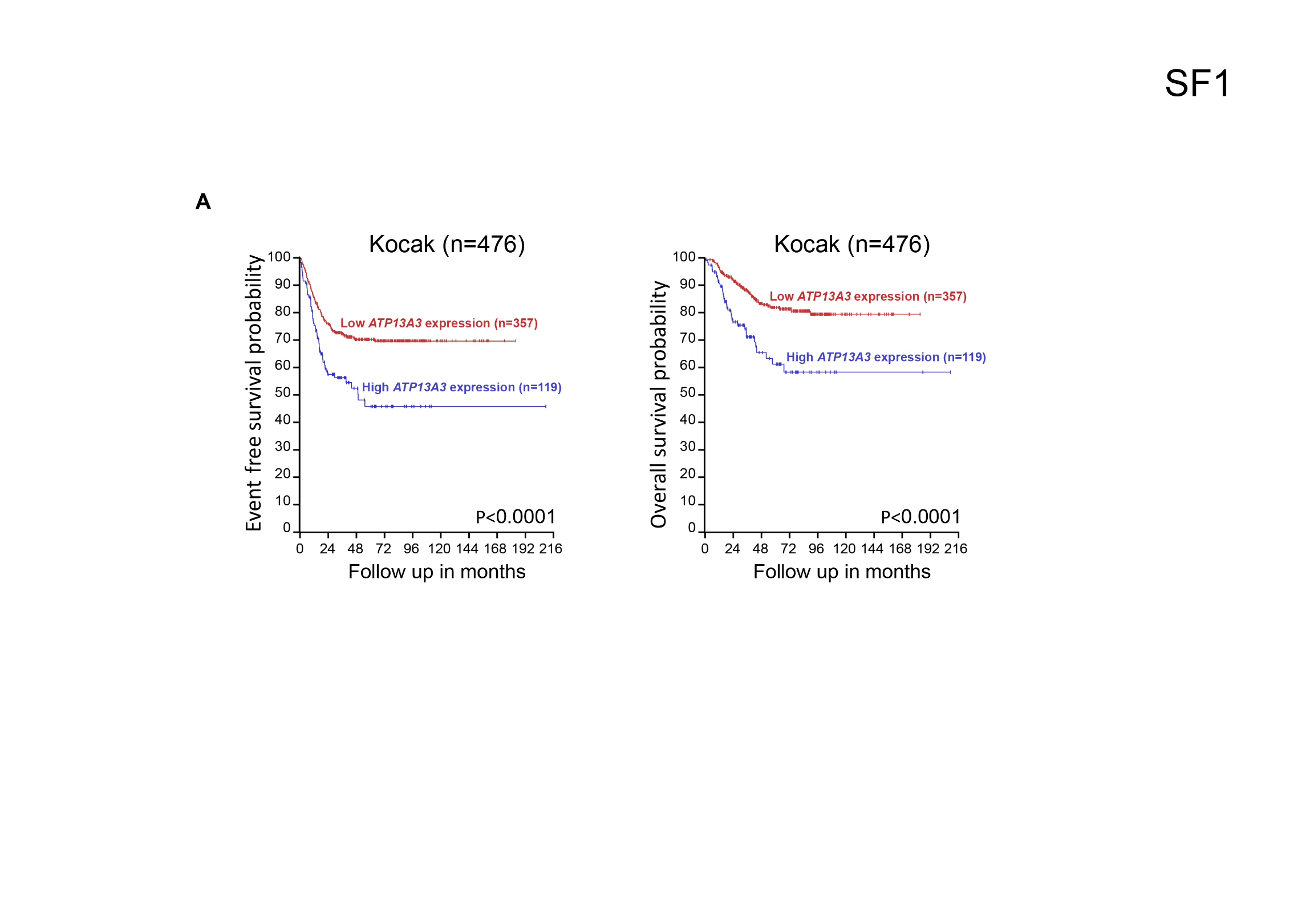
**Supplementary Figures**


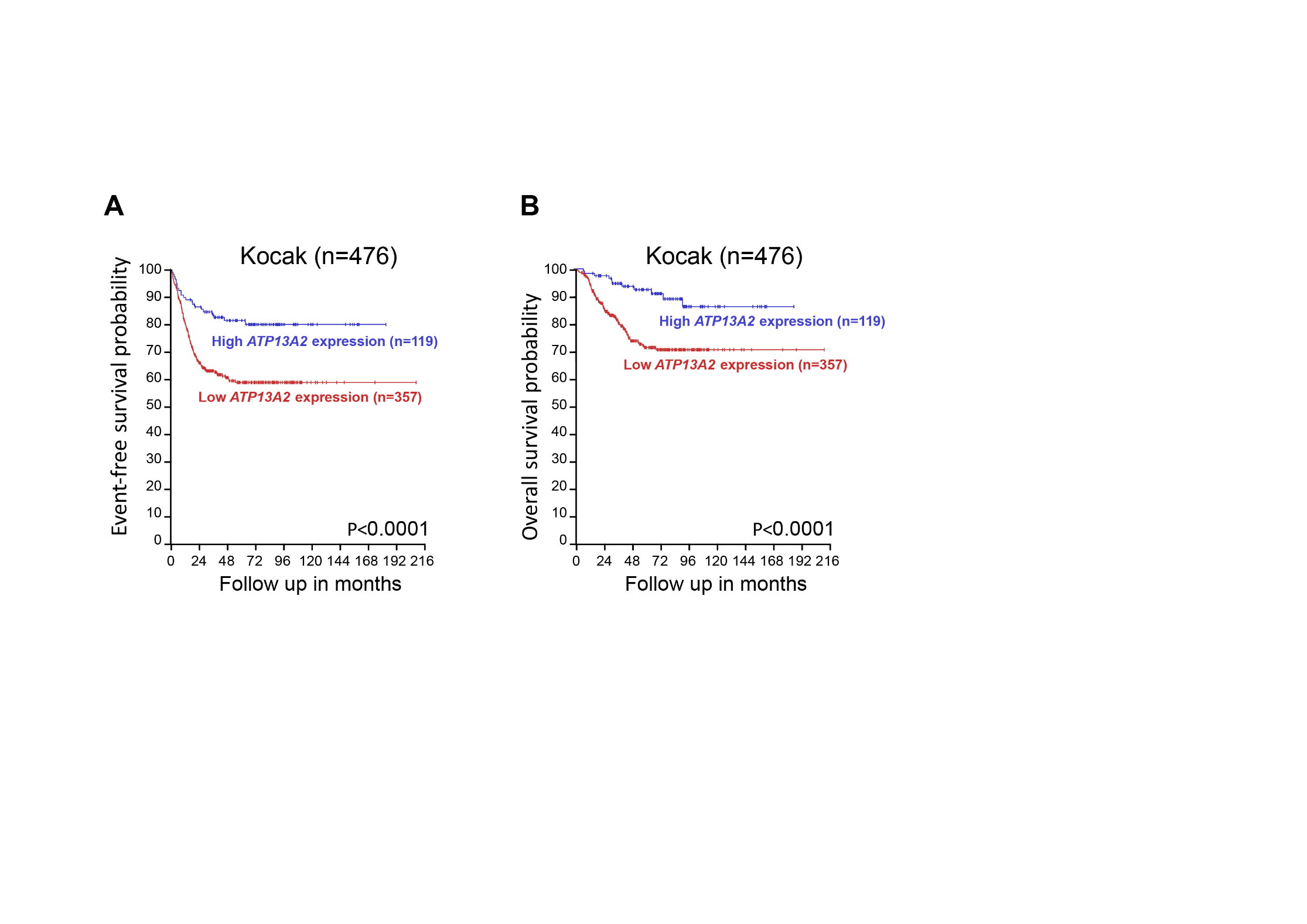
**Supplementary Figure 1: High expression of *ATP13A3* is associated with significantly worse outcome in neuroblastoma patients. (A – B)** High expression level of *ATP13A3* is associated with worse event-free and overall survival in the Kocak (n=405) patient cohort. Patients were dichotomized around the upper quartile (UQ). Log-rank test was used to compare survival curves.

**Supplementary Figure 2: High expression of *ATP13A2* is associated with a better prognosis in neuroblastoma patients. (A – B)** Event free and overall survival curves for high and low *ATP13A2* expression in the Kocak (n=649) cohort. Log-rank test was used to compare survival curves.


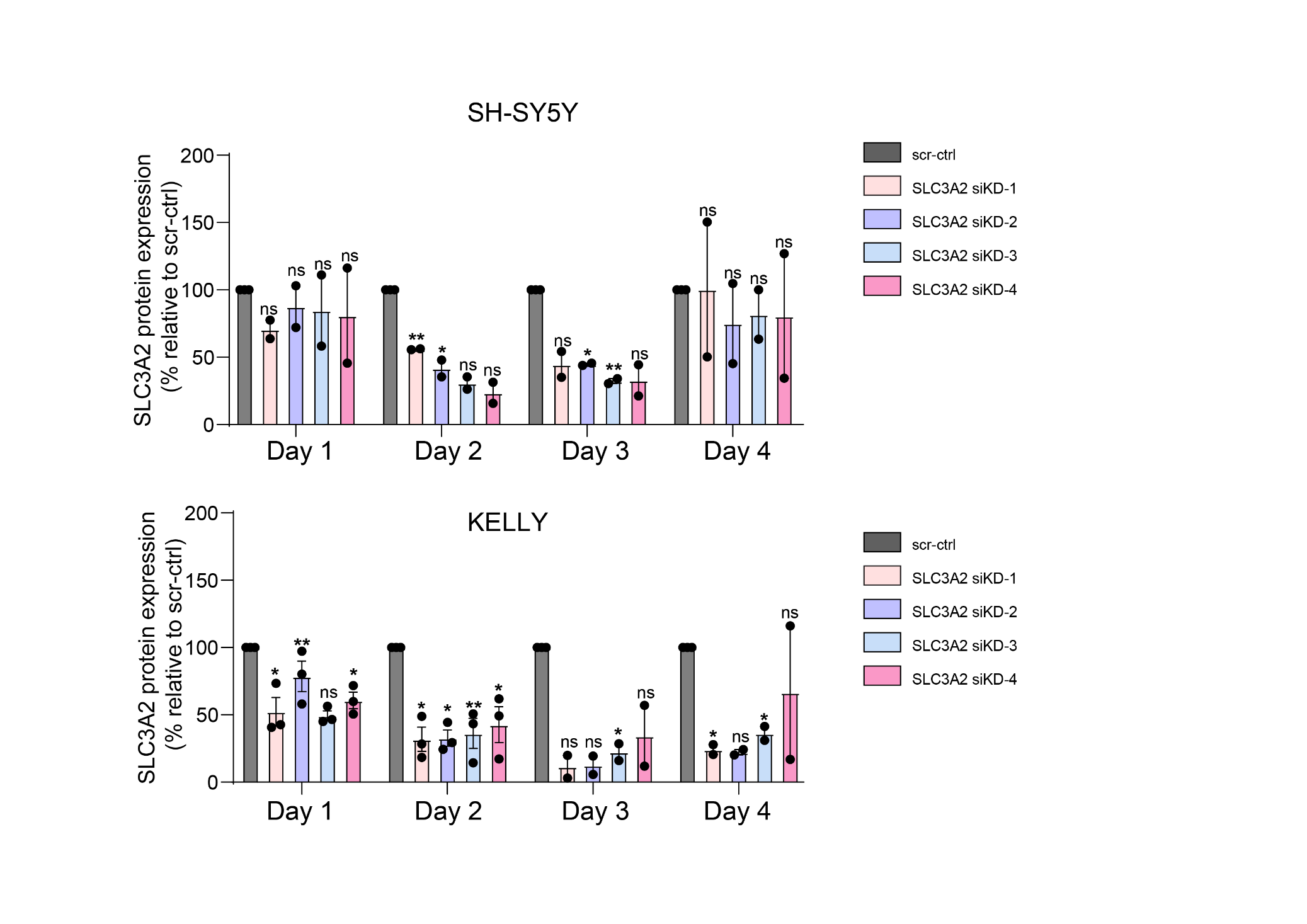
**Supplementary Figure 3: Protein expression of SLC3A2 in SH-SY5Y and KELLY cells at several time points after siRNA-mediated silencing with four different siRNAs.** Graph depicts at least two replicates for all conditions.

**Supplementary Table 1:** **Metabolomics data displayed as absolute metabolite concentrations in SH-SY5Y cells overexpressing ATP13A3 WT or the catalytically dead ATP13A3 D498N mutant.**


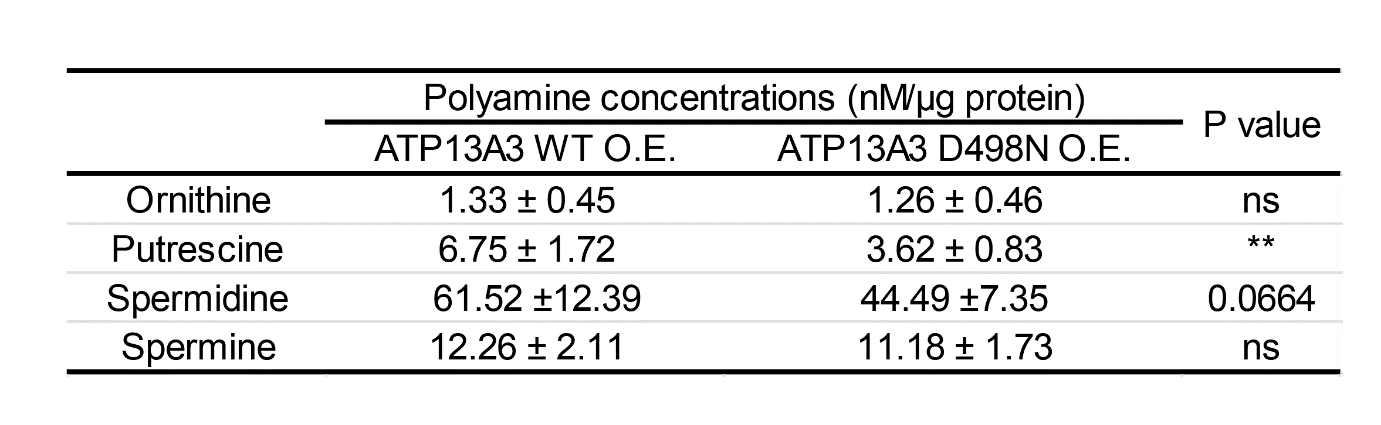


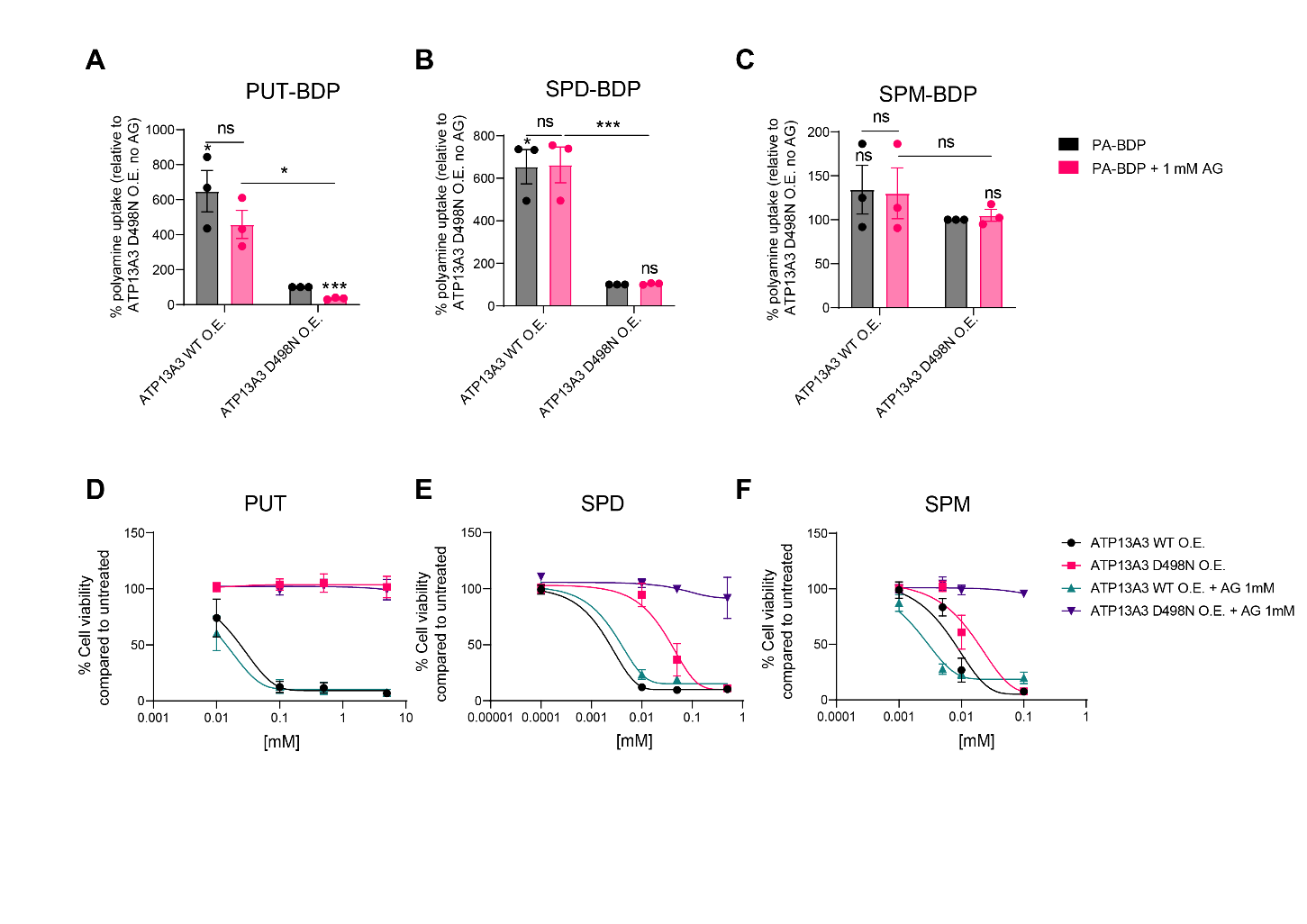

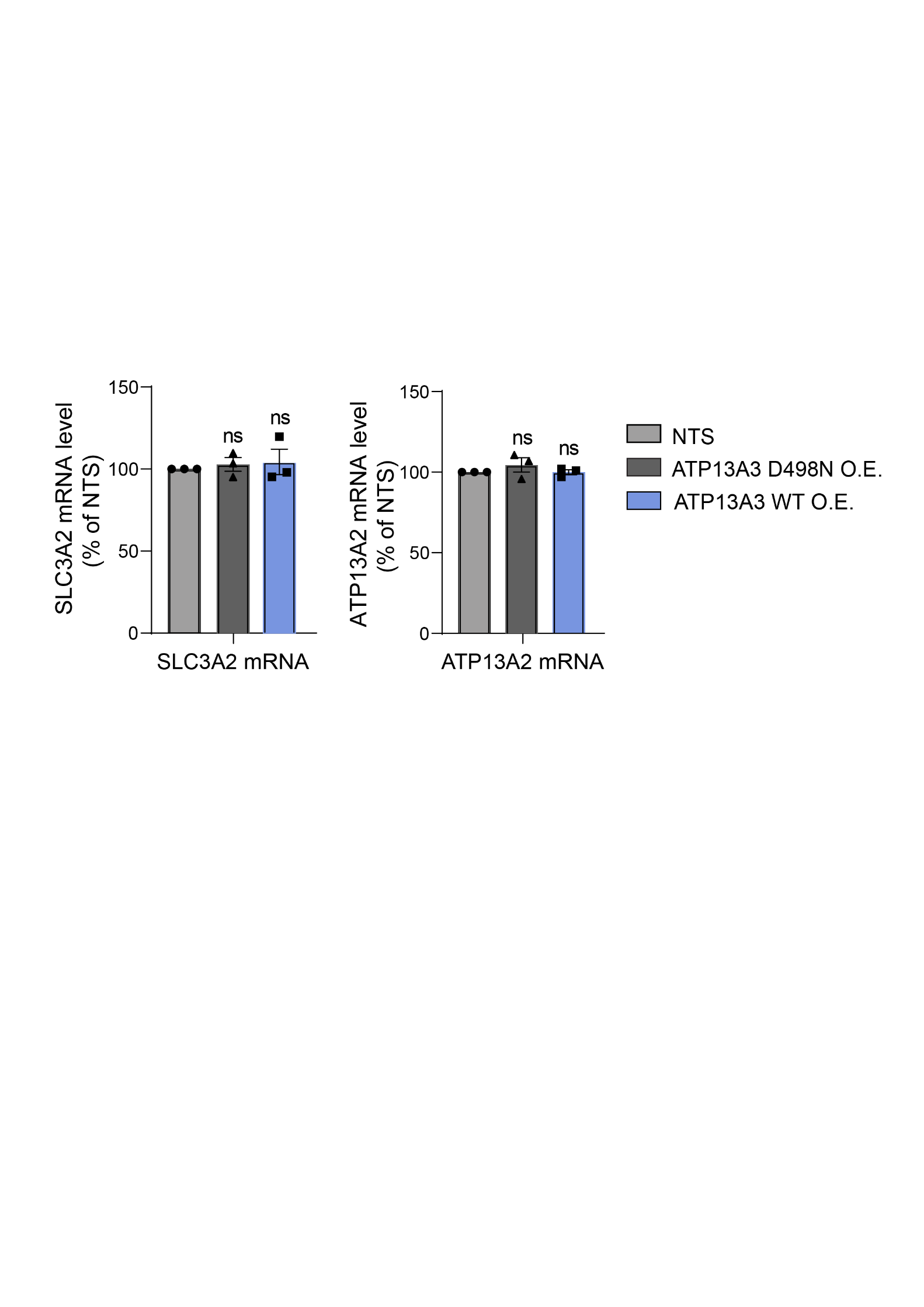
**Supplementary Figure 4: qPCR analysis showing that *SLC3A2* (left) or *ATP13A2* (right) expression does not change upon the overexpression of ATP13A3 WT or D498N (dead mutant) in SH-SY5Y cells.** One sample t-test was used to compare the gene expression in the ATP13A3 overexpression cells relative to that of non-transfected control (NTS) cells (100%). All graphs depict mean ± SEM of three independent biological repeats.

**Supplementary Figure 5: Effect of amino guanidine addition on polyamine uptake and toxicity. (A – C)** Addition of 1 mM aminoguanidine (AG) does not significantly influence the relative uptake window of BODIPY (BDP)-labelled PUT, SPD or SPM in SH-SY5Y cells overexpressing ATP13A3 WT and ATP13A3 D498N. One sample t-test was used to compare mean uptake levels relative to untreated SH-SY5Y cells overexpressing ATP13A3 D498N (100%). One-way ANOVA followed by Tukey’s multiple comparisons was used to compare mean uptake levels for all other multiple comparisons. Graphs depict mean ± SEM of three independent biological replicates. **(D – F)** Increased sensitivity to polyamine toxicity upon ATP13A3 WT overexpression is not due to extracellular polyamine oxidases. No significant difference was seen in PUT, SPD or SPM toxicity for SH-SY5Y cells overexpressing ATP13A3 WT with 1 mM aminoguanidine supplementation. All graphs depict mean ± SEM of three independent biological repeats.


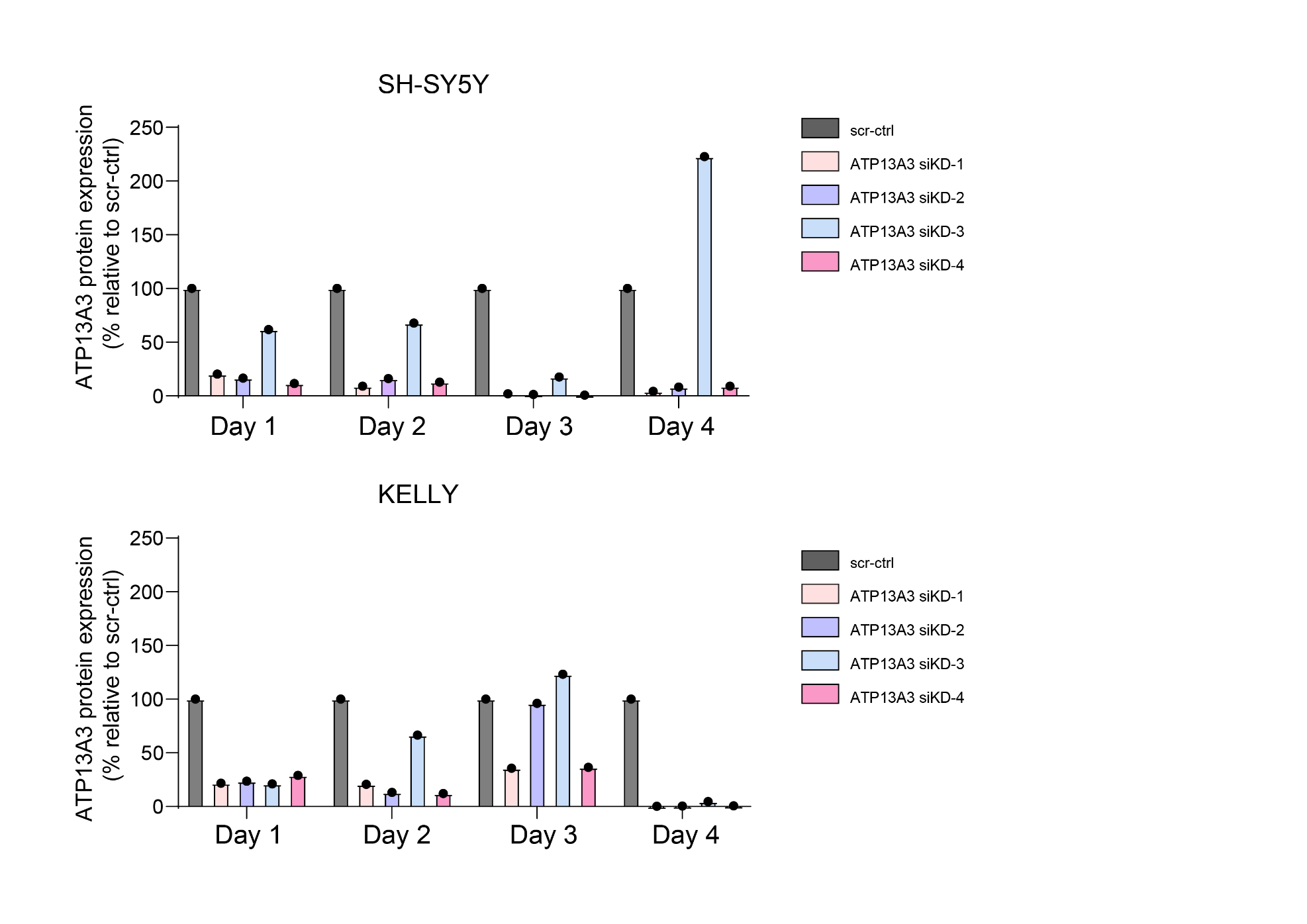


**Supplementary Figure** **6: siRNA-mediated ATP13A3 silencing decreased ATP13A3 protein expression levels in KELLY and SH-SY5Y cells from 24 h after transfection for three (siKD-1, siKD-2, siKD-4) out of four siRNAs.**


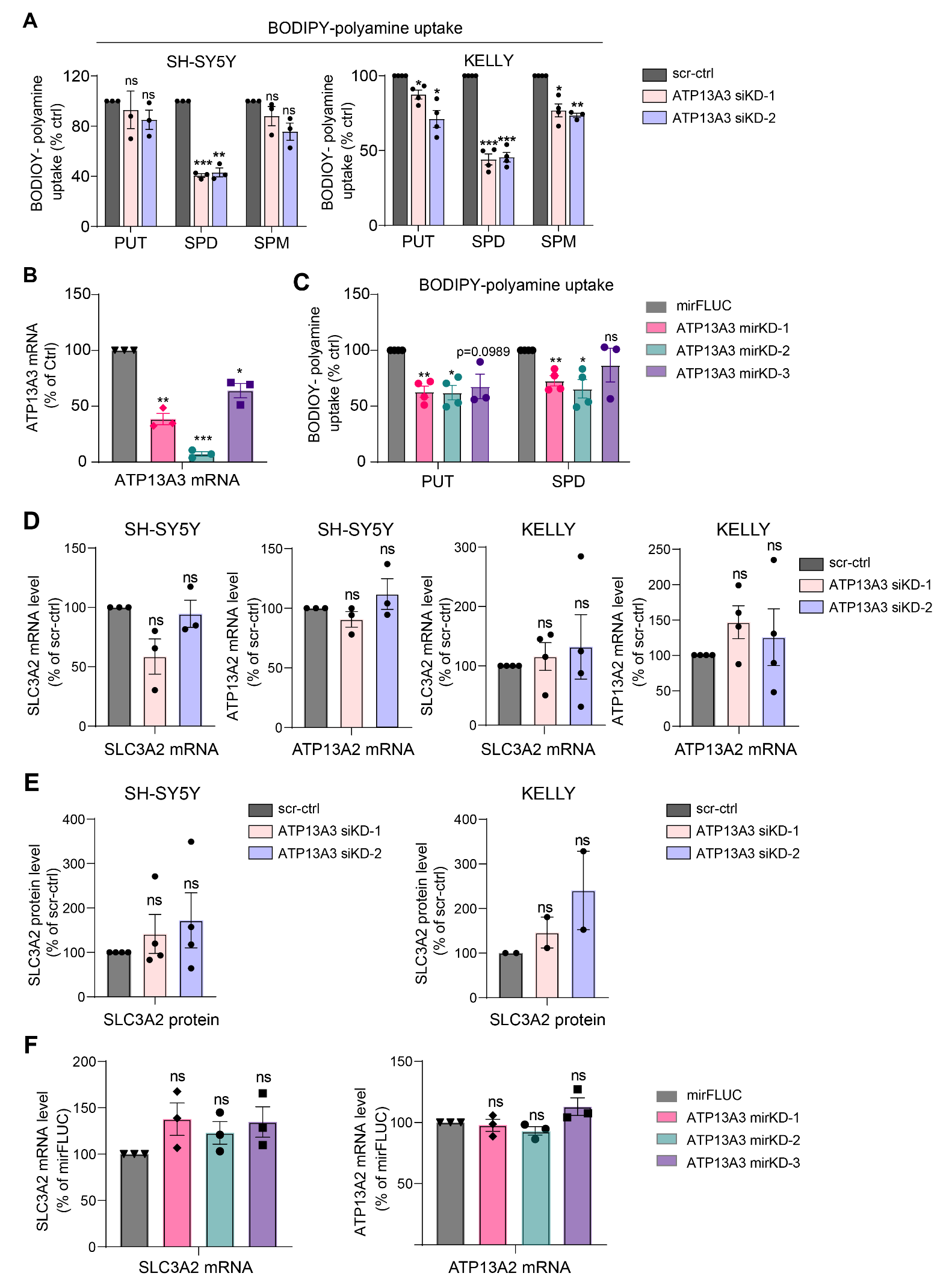


**Supplementary Figure** **7:** **Impact of ATP13A3 silencing on polyamine uptake and polyamine transporter expression in neuroblastoma cells. (A)** Transient siRNA-mediated ATP13A3 knockdown inhibits BDP-polyamine uptake in SH-SY5Y and KELLY cells at 48h. Graphs depict mean ± SEM of at least three independent biological replicates. **(B)** qPCR analysis showing *ATP13A3* mRNA levels obtained from SH-SY5Y cells with stable miRNA-based knockdown of ATP13A3. **(C)** Stable ATP13A3 knockdown in SH-SY5Y cells reduces the cellular uptake of PUT-BDP and SPD-BDP. Graphs depict mean ± SEM of at least three independent biological replicates. **(D)** *SLC3A2* or *ATP13A2* qPCR expression data upon knockdown of ATP13A3 for 48 h in SH-SY5Y and KELLY cells. All graphs depict mean ± SEM of at least two independent biological repeats**. (E)** SLC3A2 protein expression data upon knockdown of ATP13A3 for 48 h in SH-SY5Y and KELLY cells. Graphs depict mean ± SEM of at least two independent biological repeats (n=4 for SH-SY5Y and n=2 for KELLY)**. (F)** Stable ATP13A3 knockdown in SH-SY5Y cells does not affect the mRNA expression levels of *SLC3A2* or *ATP13A2*. Graphs depict mean ± SEM of three independent biological replicates. One sample t-test was used in A-E to establish the significance of the change in uptake or mRNA/protein expression level upon ATP13A3 silencing relative to control cells (mirFLUC or scr-ctrl; 100%).


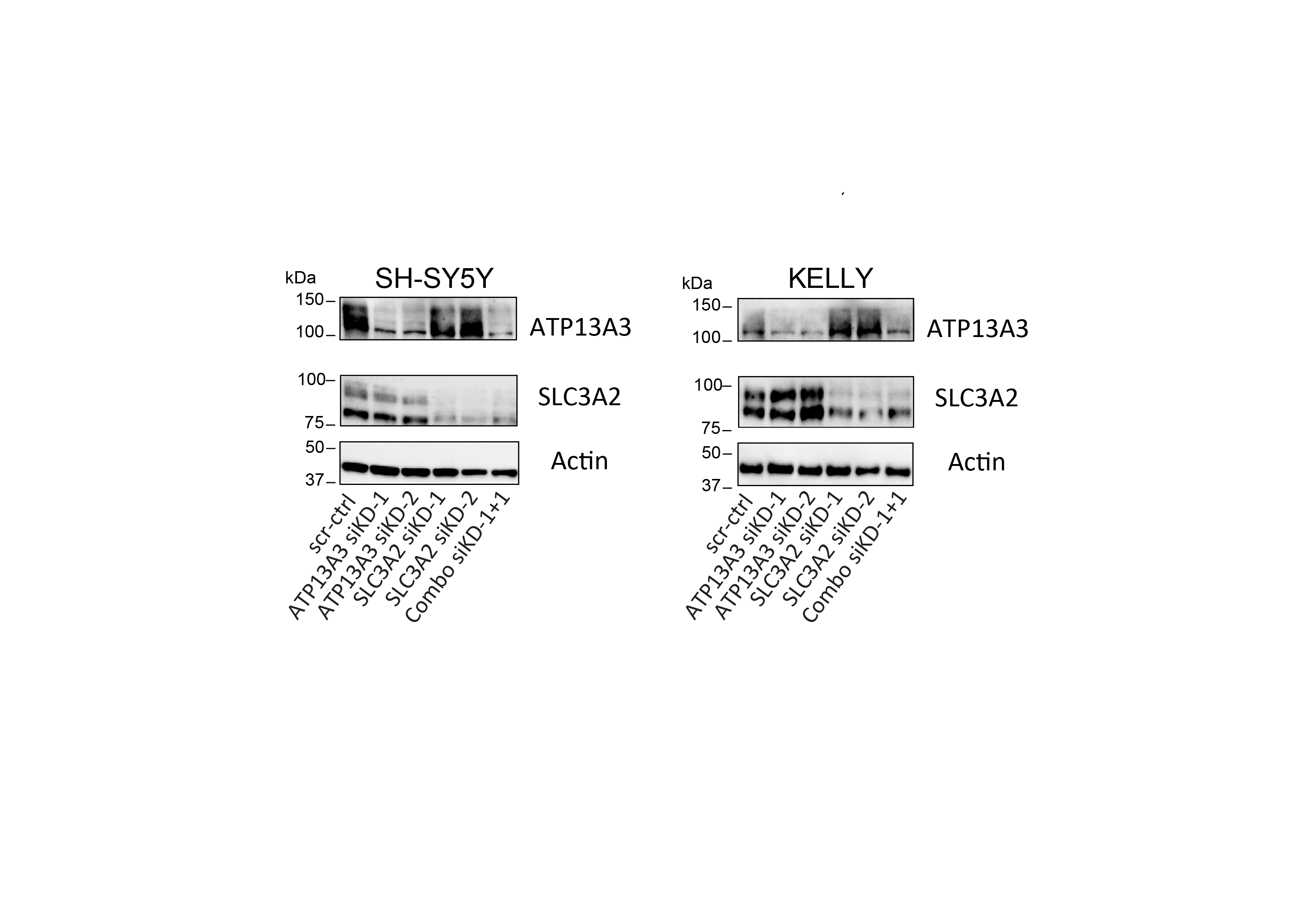


**Supplementary Figure** **8:** Western blots confirming siRNA-mediated knockdown of either ATP13A3, SLC3A2 or both compared to scrambled siRNA controls (scr-ctrl) in SH-SY5Y and KELLY cell lines. Graphs are representative for three independent experiments.


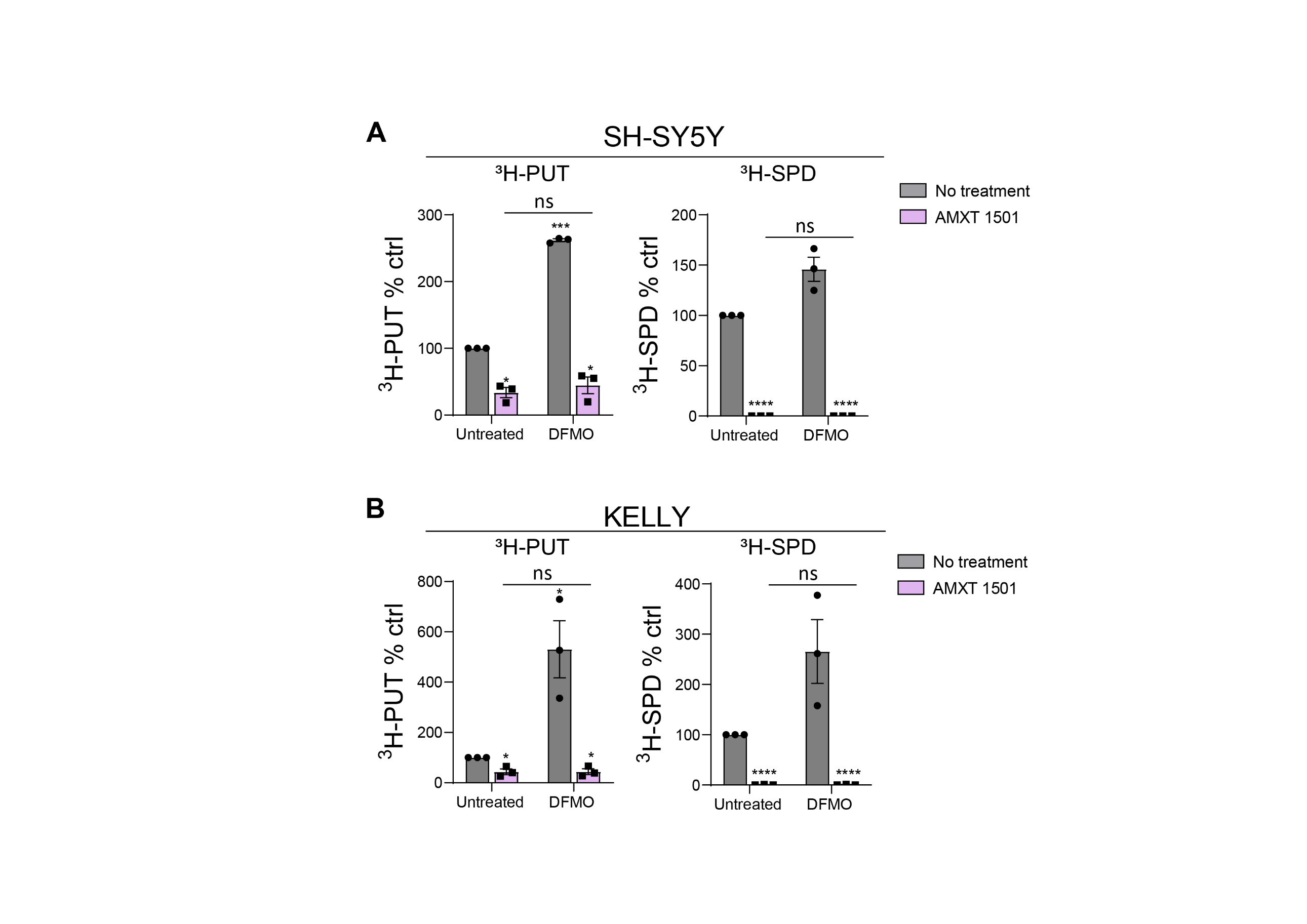


**Supplementary Figure 9:** Overnight pre-treatment with 1 µM AMXT 1501 inhibits baseline and DFMO-induced polyamine uptake in **(A)** non-*MYCN*-amplified and **(B)** *MYCN*-amplified neuroblastoma cells. One sample t-test was used for comparing mean uptake levels relative to untreated cells (stars above bars) and one-way ANOVA is used for other group comparisons (as indicated by stars above lines). Graphs depict mean ± SEM of three independent biological replicates.


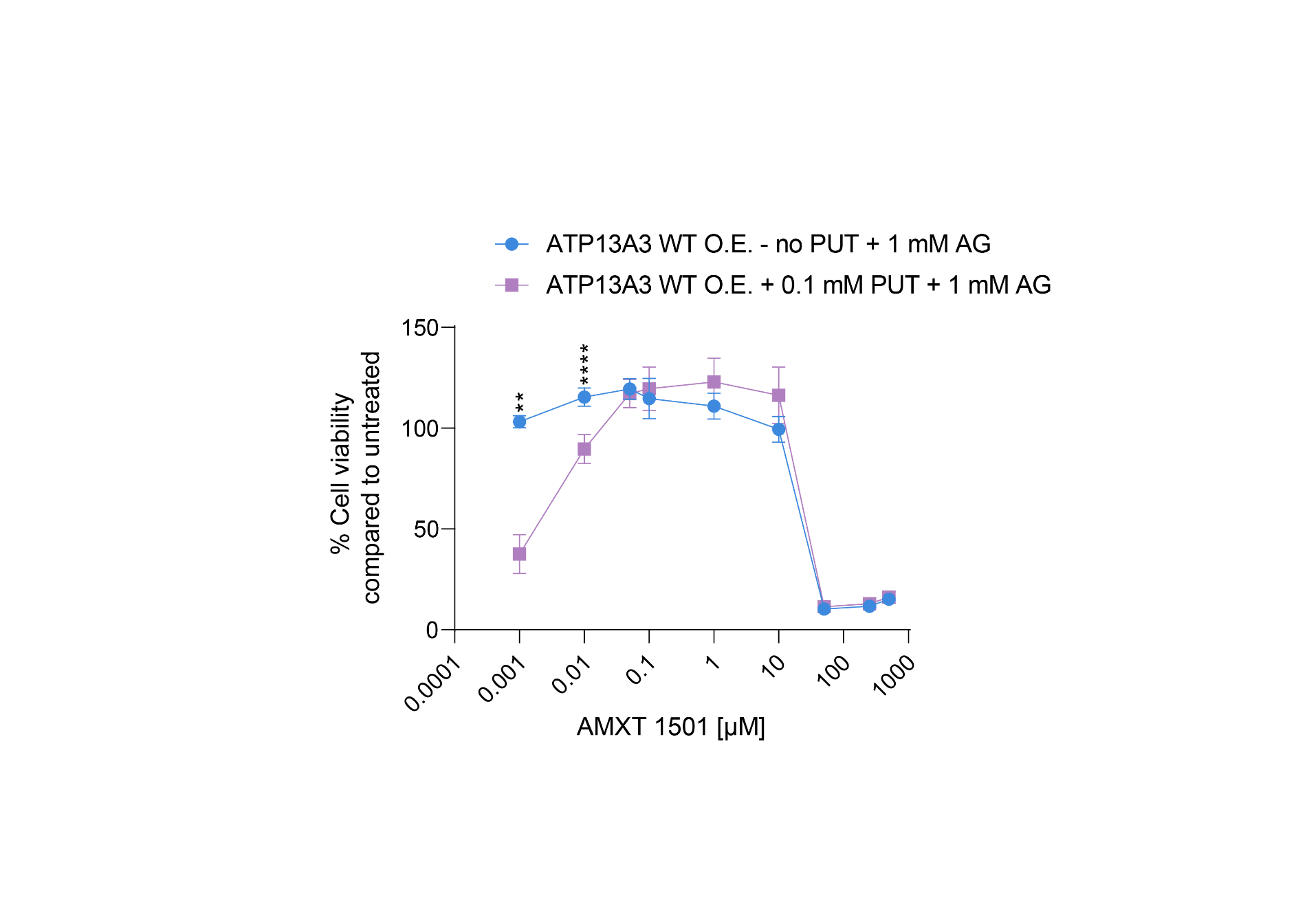


**Supplementary Figure** **10:** Cytotoxicity assay, using the MUH reagent to assess cell viability, showing the window of efficacy for AMXT 1501 in SH-SY5Y cells overexpressing ATP13A3 WT in the presence of 1 mM aminoguanidine. Graphs depict mean ± SEM of three independent biological replicates. Two-way ANOVA was used to compare dose response curves.


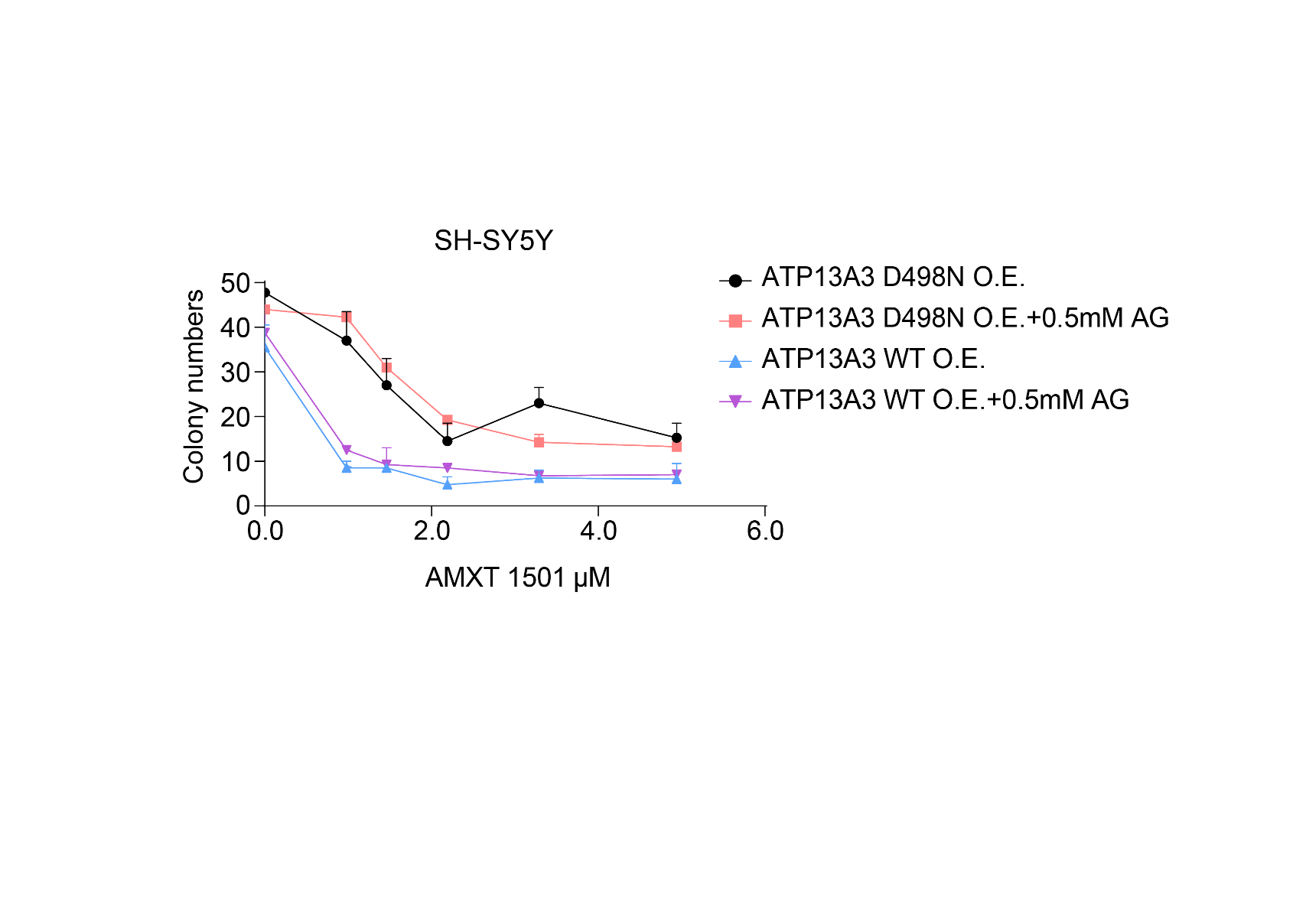
**Supplementary Figure 11:** The addition of 1 mM aminoguanidine (AG) does not affect the inhibitory effect of AMXT 1501 on colony formation of SH-SY5Y cells overexpression ATP13A3**.** Graphs depict mean ± SEM of two independent biological replicates.


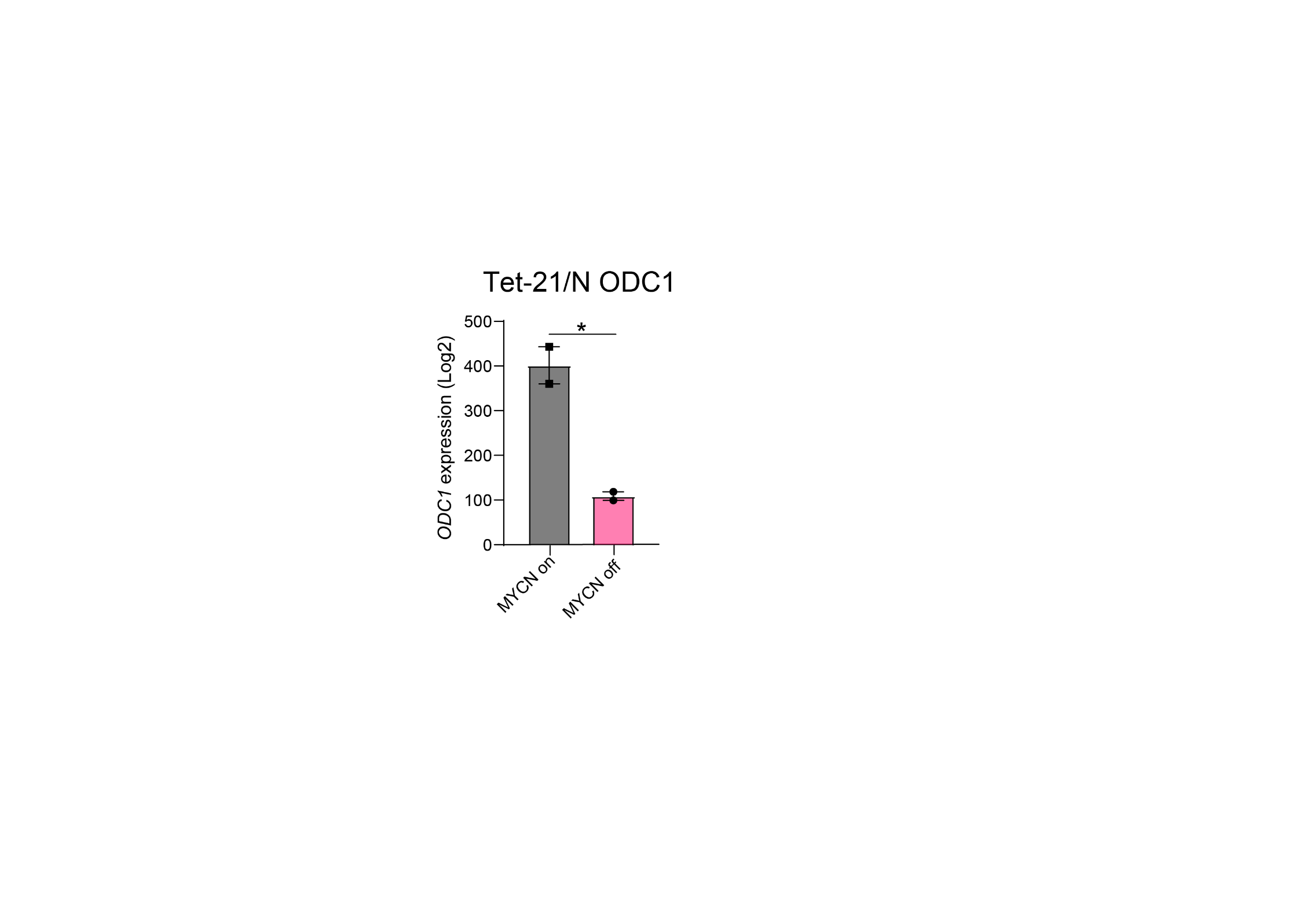
**Supplementary Figure** **12: MYCN silencing decreases *ODC1* expression in Tet-21/N cells**. mRNA expression of *ODC1* in Tet-21/N cells was determined after 72 h of doxycycline treatment. Gene expression data were obtained from the publicly available database (Accession no. GSE221103). Student t-test was used for comparing expression levels of MYCN on cells and MYCN off cells. Graphs depict the mean gene expression ± SEM of two biological replicates.


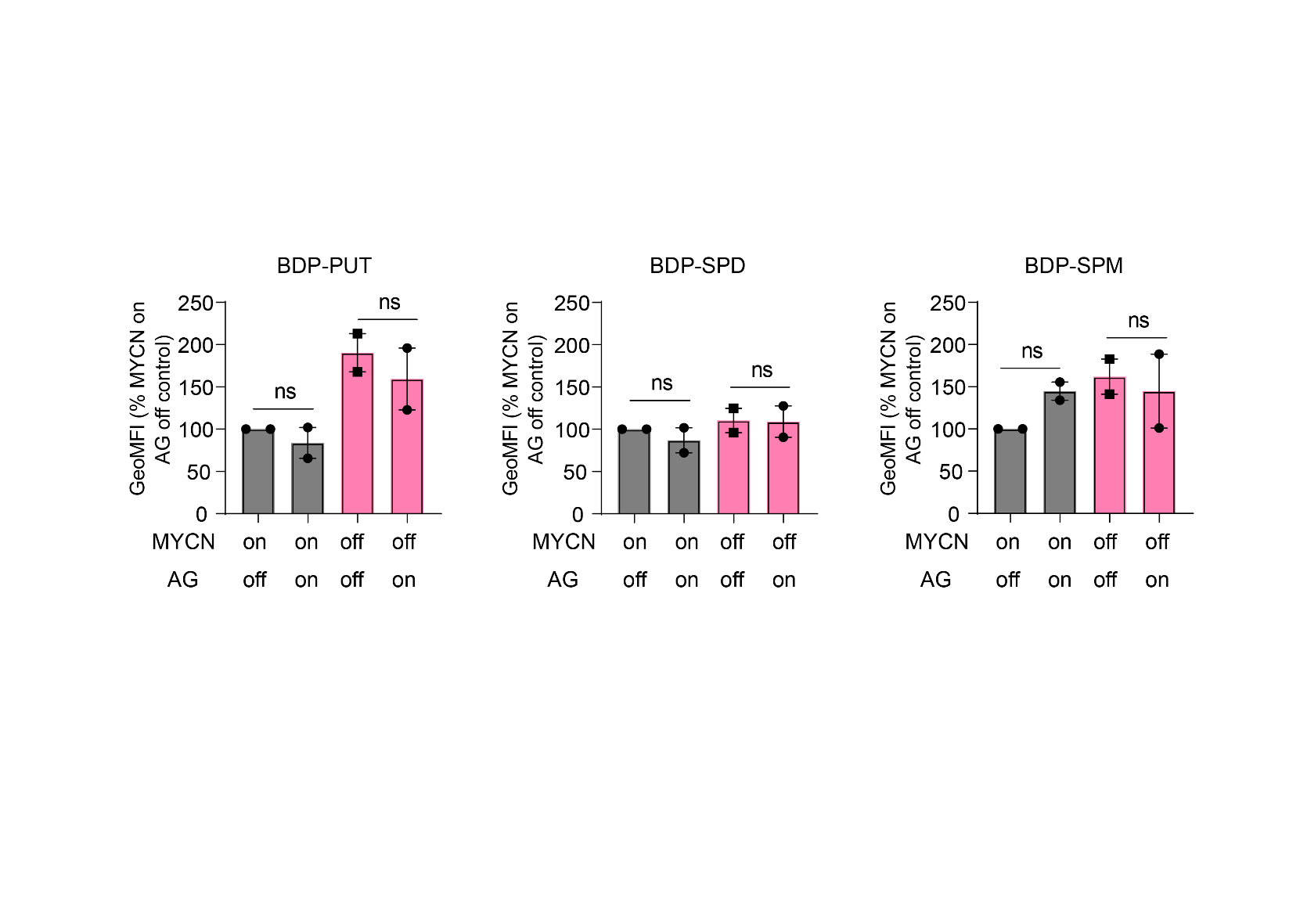


**Supplementary Figure 13:** Uptake of BODIPY-labelled polyamines in Tet-21/N cells with or without MYCN expression in the presence and absence of 1 mM aminoguanidine (AG). Polyamine uptake is measured by geometric mean fluorescence intensities (GeoMFI), relative to the MYCN on and AG off conditions (100%). One sample t-test was used to compare the difference between uptake levels and the no AG and MYCN on expression ctrl (100%), as indicated with the non-significance (ns) on the bar. The other comparisons between groups were tested with one-way ANOVA. Graphs depict mean ± SEM of two independent biological replicates.
